# Targeting the splicing factor NONO inhibits GBM progression through GPX1 intron retention

**DOI:** 10.7150/thno.72248

**Published:** 2022-07-11

**Authors:** Xu Wang, Mingzhi Han, Shuai Wang, Yanfei Sun, Wenbo Zhao, Zhiyi Xue, Xiangjun Liang, Bin Huang, Gang Li, Anjing Chen, Xingang Li, Jian Wang

**Affiliations:** 1 Department of Neurosurgery, Qilu Hospital, Cheeloo College of Medicine and Institute of Brain and Brain-Inspired Science, Shandong University, Jinan, 250012, China; 2 Jinan Microecological Biomedicine Shandong Laboratory, Jinan, 250117, China and Shandong Key Laboratory of Brain Function Remodeling, Jinan, 250012, China; 3 Medical Integration and Practice Center, Cheeloo College of Medicine, Shandong University, Jinan, 250012, China; 4 University of Pittsburgh Medical Center, Hillman Cancer Center, Pittsburgh, PA, USA; 5 Department of Biomedicine, University of Bergen, Jonas Lies vei 91, 5009 Bergen, Norway

**Keywords:** Glioblastoma multiforme, NONO, mRNA splicing, GPX1, Auranofin

## Abstract

**Background:** Splicing factors are essential for nascent pre-mRNA processing and critical in cancer progression, suggesting that proteins with splicing functions represent potential molecular targets for cancer therapy. Here, we investigate the role of splicing factors in glioblastoma multiforme (GBM) progression and the possibility of targeting them for the treatment of the disease.

**Methods:** The TCGA and CGGA public databases were used to screen for differentially expressed mRNA splicing factors. Immunohistochemistry and qRT-PCR were used to analyze the expression of non-POU domain-containing octamer-binding protein (NONO), a Drosophila behavior human splicing (DBHS) protein. Knockdown/overexpression of NONO with siRNA and lentiviral expression constructs was used to examine cell growth, apoptosis, and invasion in GBM cells. RNA sequencing was used to identify potential downstream molecular targets of NONO. RIP-PCR and RNA pulldown were used to determine the interaction between NONO and pre-mRNA. JC-1 staining and the seahorse assay were performed to assess redox homeostasis.

**Results:** Expression of NONO was increased in GBM samples and associated with poor survival in patients (*P* = 0.04). Knockdown of NONO suppressed GBM growth, and overexpression of NONO promoted GBM tumorigenesis *in vitro* and *in vivo*. RNA sequencing-based transcriptomic profiling confirmed that knockdown of NONO in U251 and P3 cells resulted in global intron retention of pre-mRNA and led to abnormal splicing of specific pre-mRNAs for *GPX1* and *CCN1*. NONO bound to a consensus motif in the intron of *GPX1* pre-mRNA in association with another DBHS protein family member, PSPC1. Knockdown of NONO impaired tumor growth, invasion, and redox homeostasis through aberrant splicing of *GPX1*. Finally, Auranofin, a small molecule inhibitor of NONO, suppressed GBM tumor growth in an orthotopic xenograft model in mice.

**Conclusions:** We demonstrated that intron retention was a critical alternative RNA splicing event to occur in GBM progression, and that NONO was a key regulator of mRNA splicing in GBM. Targeting NONO represents a novel, potential therapeutic strategy for GBM treatment.

## Introduction

The hyperactivation of transcription is a common feature of various cancers producing an increase in nascent mRNA which requires processing 1. Pre-mRNA splicing is a post-transcriptional process used to generate multiple mRNA isoforms from a single gene. As the mRNA determines the protein sequence and function, the splicing process has the potential to expand the variety of encoded proteins. Pre-mRNA expression is regulated by transcription, and the relative abundance of specific mRNA depends on the splicing context 2. RNA sequencing has indicated that greater than 90% of human genes encoding proteins undergo pre-mRNA splicing to remove introns, and intron retention is a common type of abnormal splicing 3-5. Recent studies have reported numerous aberrant splicing events that influence cancer progression, including glioblastoma multiforme (GBM). Therefore, splicing factors are potentially therapeutic targets for cancer therapy, including in the treatment of GBM. In fact, we have previously identified USP39 as a dysregulated splicing factor for precursor messenger RNA (pre-mRNA) maturation in GBM 6. However, the underlying roles of these tumor-specific events and the abnormal mechanisms for splicing, and especially for intron retention in GBM, remain unclear.

Previous studies have reported that certain RNA binding proteins (RBPs) with splicing functions are dysregulated and promote the development of cancer 7, 8. Non-POU domain-containing octamer-binding protein (NONO) is an RBP and also belongs to the Drosophila behavior human splicing (DBHS) family. Other members of the DBHS family include the paralogs splicing factor, proline- and glutamine-rich (SFPQ) and paraspeckle component 1 (PSPC1) 9. These predominantly nuclear proteins have two RNA-recognition motifs (RRM) and are components of subnuclear body-paraspeckles. The paraspeckle complex is known to regulate DNA repair and RNA metabolism, including splicing, stabilization and export 10.

Similar to many RBPs, NONO exerts its various functions through multiple mechanisms, and participates in different physiological and pathological conditions. For example, NONO is essential for cGAS-mediated innate immune activation and directly binds the viral capsid in the immunity pathway 11. The protein is also recruited to repair DNA damage 12 and suppresses telomere instability 13. Apart from these roles, NONO is required in transcriptional and post-transcriptional regulation at different stages. The Drosophila ortholog of NONO, NonA, facilitates pre-mRNA splicing and upregulates *cpx* expression. However, the mechanism of NonA in pre-mRNA processing is unclear in this study 14. NONO deficiency interferes with TET1-associated transcription through epigenetic mechanisms in mice 15. NONO also coordinates pre-mRNA processing of metabolic genes, especially the removal of introns 16. However, the function of NONO requires further elaboration in RNA splicing and the development of cancer, especially in GBM, which has been until now largely unexplored.

In this study, we found the mRNA splicing protein NONO to be overexpressed in human gliomas based on analysis of expression data from publicly available databases, The Cancer Genome Atlas (TCGA) and the Chinese Glioma Genome Atlas (CGGA). We demonstrate that NONO promotes GBM proliferation and invasion through splicing of specific pre-mRNAs such as *GPX1*. Splicing of the *GPX1* pre-mRNA required a specific functional domain of NONO and interaction with other DBHS protein family members. Knockdown of NONO altered the state of GBM redox homeostasis which was rescued with ectopic expression of GPX1. Finally, we tested Auranofin, a small molecule inhibitor previously reported to target NONO 17, in a xenograft model for GBM. Auranofin thus provides a promising candidate molecule for the treatment of GBM.

## Materials and Methods

### Ethics statement and clinical glioma tumor specimens

Approval for the protocols in the study was granted by the Ethics Committee of Qilu Hospital of Shandong University (DWLL-2021-041). This study was conducted in full adherence to relevant regulations and guidelines. Human glioma tissue samples were obtained from surgeries performed on patients at Qilu Hospital. Non-neoplastic brain tissue samples were obtained from patients requiring surgery for traumatic brain injury events. All patients enrolled provided written informed consent.

### Immunohistochemistry (IHC)

Sections (5 μm) were cut from paraffin-embedded tissues and incubated with primary antibody. Detection was performed through incubation with the species appropriate secondary antibody conjugated to horse radish peroxidase and the substrate DAB. The following primary antibodies were used: rabbit anti-NONO (ab70335, 1:200; Abcam; Waltham, MA, USA), rabbit anti-Ki67 (GB13030-2, 1:1,000; Servicebio; Wuhan, China) and rabbit anti-GPX1 (ab22604, 1:200; Abcam). Staining was evaluated independently to determine the histological score according to the proportion of positive staining cells and staining intensity.

### Cell culture

The A172, LN229, U251, U118, and U87 cell lines were purchased from ATCC, and cultured in Dulbecco's modified Eagle medium (Thermo Fisher Scientific; Waltham, MA, USA) supplemented with 10% fetal bovine serum (FBS; Thermo Fisher Scientific). Patient-derived GBM stem-like cells (GSCs) P3, BG5 and BG7 were previously isolated and characterized from GBM surgical specimens 18, 19. GSCs were cultured in Neurobasal medium (Gibco/Thermo Fisher Scientific) containing 2% B-27 Neuro Mix (Thermo Fisher Scientific), 20 ng/mL epidermal growth factor (EGF; PeproTech; East Windsor, NJ, USA), and 10 ng/mL basic fibroblast growth factor (bFGF; PeproTech). Normal human astrocytes (NHA) and NHAs transfected with human papillomavirus 16 E6/E7 and human TERT (immortalized NHA-ET) were obtained from Lonza (Walkersville, MD, USA) and cultured in Astrocyte Medium (ScienCell; Carlsbad, CA, USA) supplemented with the Astrocyte Growth Medium BulletKit (ScienCell). Cells were treated with small molecules Auranofin (Selleck; Houston, TX, USA), Madrasin (MedChemExpress (MCE); Beijing, China) or cycloheximide (Cell Signaling Technology; Danvers, MA, USA) for 48 h.

### qRT-PCR

Total RNA was extracted using the RNA-Quick Purification Kit (ES Science; Shanghai, China) with DNase treatment and reverse-transcription was performed with the ReverTra Ace qPCR RT Kit (Toyobo; Osaka, Japan). cDNA was amplified using TB Green on the Roche Light Cycler 480 for quantification (Roche; Indianapolis, IN, USA). The relative expression levels of mRNA were normalized to glyceraldehyde-3-phosphate dehydrogenase (GAPDH). Sequences of the primers used are shown in Table S1.

### siRNA and short hairpin RNA (shRNA) transfection

Both transient and stable transfections were performed with Lipofectamine 2000 reagent (ThermoFisher Scientific) following the manufacturer's instructions. For siRNA experiments, cells were transfected with 100 pmol of siRNA (GenePharma; Shanghai, China) for 48 h. Lentiviral supernatants for stable expression were harvested 48 h after transfection of HEK293T cells with the lentiviral packaging plasmids, psPAX2 and pCMV-VSV-G, and the lentiviral expression construct. Target cells were cultured with supernatant for 24 h and selection with puromycin began after 48 h. The sequences of the siRNAs targeting NONO, GPX1, SFPQ, and PSPC1 were the following: siNONO-1: 5'-GCCAGAAUUCUACCCUGGAAA-3'; siNONO-2: 5'-GCAUUCCUGAAGUCUCUAA-3'; siGPX1-1: 5'-GCAAGGUACUACUUAUCGAGA-3'; siGPX1-2: 5'-GCAUCAGGAGAACGCCAAGAA-3'; siSFPQ-1: 5'-GUACGAAUAUUCUCAGCGA-3'; siSFPQ-2: 5'-GGAAGAUGCCUAUCAUGAA-3'; siPSPC1-1: 5'-CUUGACUGUCAAGAACCUU-3'; siPSPC1-2: 5′-GCUGCUAGAGCAAGCAUUU-3'; siNC: 5'-UUUUCCGAACGUGUCACGUTT-3'. Expression constructs for shNC, shNONO, GPX1, FLAG-NONOwt, FLAG-NONO RRM1 mutation (RRM1mut), FLAG-NONO RRM2 mutation (RRM2mut), and FLAG-NONO DBHS mutation (DBHSmut) were purchased from Obio Technology (Shanghai, China). pRL-TK reporter plasmids were purchased from GenePharma. Promoter regions were selected as those located 2000 bp upstream of the beginning of the gene, and the promoter region of *GPX1* and *CCN1*, respectively, were cloned into the pRL-TK reporter vectors. After 48 h of transfection, luciferase activity was determined using a luciferase reporter gene assay kit (Beyotime; Shanghai, China).

### Cell proliferation and colony forming assays

Cell viability was measured using CCK-8 (Beyotime) following the manufacturer's protocol. Briefly, cells were seeded in 96-well plates, cultured for 24 h, and incubated with CCK-8 at 37 °C for 1 h. Absorbance was measured at 450 nm and the time point of the transfection was considered as 0 h. For colony forming assays, treated cells were seeded into 6-well plates (1 × 10^3^ cells/well) and cultured for 2 weeks. Cells were fixed with 4% paraformaldehyde and stained with crystal violet. Colonies with over 100 cells were counted for analysis.

### Cell invasion assays

For transwell assays, after 24 h of transfection, cells (3 × 10^4^ cells/well) were seeded into the upper chambers of 12-well plates without FBS, and medium containing 15% FBS was placed in the lower chambers. Chambers were incubated at 37 °C for 24-48 h, and the cells migrating through the membranes were fixed with 4% paraformaldehyde and stained with crystal violet. Images were obtained and cells were counted. For the 3D tumor spheroid invasion assay, cell spheres were embedded in matrigel (Trevigen; Gaithersburg, MD, USA) and incubated for 72 h at 37 °C. The sphere diameter was regarded as the starting point for quantification.

The GBM-brain organoid co-culture invasion *ex vivo* system, such as the culture of 18-day rat fetal brain organoids, was performed as previously described 20. GFP-transfected GBM cells were cultured to generate glioma spheroids and then co-cultured with mature brain organoids for 72 h. GBM cell invasion images were captured under confocal microscopy (Leica TCS SP8; Wetzlar, Germany).

### Flow cytometry

For cell cycle analysis, cells were harvested, fixed with 75% ethanol for 48 h at 4 °C and stained with propidium iodide (PI; BD Biosciences; Franklin Lakes, NJ, USA) for 15 min. For the detection of apoptosis, cells were rinsed with PBS, resuspended in staining buffer and incubated with Annexin V-FITC and PI (BD Biosciences) for 15 min. The mitochondrial membrane potential was determined with the Mitochondrial Membrane Potential kit (Beyotime). All the cells were assessed on a C6 flow cytometer (BD Biosciences) and the data were analyzed with FlowJo software (V10, BD Biosciences).

### Immunofluorescence and RNA fluorescence *in situ* hybridization

Cells were seeded on coverslips and then fixed with 4% paraformaldehyde. Immunofluorescence staining was performed to determine the subcellular localization of NONO and SC-35 with the following antibodies: NONO antibody (Abcam) and SC-35 (Sigma-Aldrich; St. Louis, MO, USA). FAM or CY3 modified FISH probes were used to detect the *GPX1* pre-mRNA and mature mRNA following the manufacturer's protocol (GenePharma).

### Immunoprecipitation (IP) and western blotting

Cells were lysed in IP lysis buffer (Pierce/ThermoFisher Scientific) containing a protease inhibitor cocktail (Sigma-Aldrich). Total lysates were incubated with anti-FLAG (14793S; Cell Signaling Technology) or anti-IgG (ab172730; Abcam) overnight at 4 °C and then mixed with Protein A/G magnetic beads (Thermo Fisher Scientific) for 2 h at room temperature. Proteins were eluted and run on SDS-PAGE for western blot analysis. The following primary antibodies were used: anti-NONO (ab70335; Abcam), anti-N-cadherin (13116S; Cell Signaling Technology), anti-CD44 (3570S; Cell Signaling Technology), anti-BCL2 (4223S; Cell Signaling Technology), anti-BAX (50599-1-Ig; ProteinTech; Wuhan, China), anti-GPX1 (ab22604; Abcam), anti-CCN1 (14479S; Cell Signaling Technology), anti-SFPQ (15585-1-AP; ProteinTech), anti-PSPC1 (ab104238; Abcam), and anti-ZEB1 (3396S; Cell Signaling Technology).

### RNA sequencing, data processing and bioinformatics analysis

Expression data and associated clinical data were downloaded from publicly available databases, the TCGA and the CGGA 21, and analyzed with the EdgeR package. The volcano plot and heatmap were obtained using the Hiplot website. Differential expression and pathway analysis was performed using gene set enrichment analysis (GSEA).

U251 and P3 mRNA sequencing (mRNA-seq) was performed by the LC Bio Corporation (Hangzhou, China). Sequence analysis was performed on the Illumina PE150 model (Illumina; San Diego, CA, USA). The sequencing depth was 2x, and 3 biological replicate samples were analyzed in each group. Differential gene expression was determined based on fold change (FC; |log2(FC)|) of > 1 and a *P* value of < 0.05. DAVID was used to perform GO analysis, and visualization was accomplished using R software. Sequencing data were viewed in Integrative Genomics Viewer (IGV). In brief, reads were aligned to the hg19 genome with HISAT2 (version 2.2.0) and sorted with samtools (version 1.9). Putative splicing events and transreads were identified using Regtools 0.2.0 and Bedtools (version 2.27.1). Splicing efficiency was determined for the 5' and 3' splice sites as follows: Efficiency = transread count/5' and 3' intron end first base coverage. The splicing efficiency of genes was calculated in R package Splicing Efficiency Analysis and Annotation (SEAA).

### RNA immunoprecipitation (RIP) assays and biotin-labeled RNA pull-down

RIP was performed using the EZ-Magna RIP RNA-Binding Protein Immunoprecipitation Kit (Merck Millipore; Burlington, MA, USA). In brief, cells were cross-linked with 1% formaldehyde and lysed with protease and RNase inhibitors. Magnetic beads preincubated with IgG or antibody specific for NONO (ab70335; Abcam) or PSPC1 (ab104238; Abcam) were incubated with lysates at 4 °C overnight. Eluted RNAs were purified and detected with qPCR. Total RNA was regarded as the input control.

Human *GPX1* pre-mRNA (sense and antisense; GenePharma) and mRNA (GenePharma) were transcribed* in vitro* using the Transcript Aid T7 High Yield Transcription Kit (ThermoFisher Scientific). mRNAs were 3' end labeled with biotin using the RNA 3' End Desthiobiotinylation Kit (ThermoFisher Scientific), and associated proteins were pulled down in coprecipitation assays and examined on western blot.

### Measurement of GPx activity, reactive oxygen species (ROS) and Seahorse XF analysis

GPx activity was detected with the total glutathione peroxidase assay kit with NADPH (Beyotime) following the manufacturer's protocol. Intracellular ROS, H_2_O_2_ and glutathione (GSH) levels were measured with the ROS Assay Kit (Beyotime), the Hydrogen Peroxide Assay Kit (Beyotime) and the GSH and GSSG Assay Kit (Beyotime), respectively, according to the manufacturer's instructions. Fluorescence intensity was measured with fluorescence microscopy (Leica) and flow cytometry (C6; BD Biosciences) using excitation and emission wavelengths of 488 nm and 525 nm, respectively.

U251 and P3 cells were seeded onto Seahorse XF 24-well plates (Seahorse Biosystems, Agilent Technologies). After NONO inhibition, the medium was replaced with assay medium (200 μL; pH 7.35) containing 2 mM L-glutamine, 1 mM pyruvate and 25 mM glucose, and the plate was incubated for 1 h at 37 °C without CO_2_. The Seahorse XF Biosystem was used to analyze the oxygen consumption rate (OCR). Oligomycin (2 μM), FCCP (1 μM) and rotenone (500 nM) were successively added to cells to determine the OCR which was normalized to protein content.

### Protein purification and surface plasmon resonance (SPR)

The pET-28a(+)-His-NONO plasmid was purchased from GenePharma. The expression vector was transfected into *Escherichia coli BL21* (TransGen Biotech; Beijing, China). The obtained strains were grown in LB medium supplemented with 0.1 mg/mL kanamycin at 37 °C with shaking at 200 rpm. Protein expression was induced with 0.5 mM isopropyl-β-D-thiogalactopyranoside (Solarbio; Beijing, China) and purification was performed with the His-Tagged Protein Purification Kit (CoWin Bio; Cambridge, MA, USA).

SPR was performed with SensiQ (The Pioneer platform, ForteBio; Freemont, CA, USA). First, the SPR chip (Hiscap biosensor, ForteBio) was activated with 1 mM NiCl_2_, and 50 μg/mL His-NONO was immobilized on the chip. Small molecule (300 µM Auranofin) binding activities were generated with the SPR system, and the binding signal was exhibited by the response (RU) value. The data were normalized to control and analyzed with Qdat (ForteBio). Binding curves were subsequently generated.

### Intracranial GBM xenografts and Auranofin treatment

Athymic nude mice (Foxn1^nu^ mut/mut; 4-week-old males; SLAC Laboratory Animal Center; Shanghai, China) were bred under pathogen-free conditions at 24 °C on a 12-h day-night cycle. Mice were randomly grouped (n = 5 per group), anesthetized, and injected with luciferase-expressing LN229, U251 or P3 cells (5 × 10^5^ cells suspended in 10 μL PBS) into the frontal lobe. The burr hole was positioned 1 mm anterior and 2 mm lateral from the anterior fontanel and the injection depth was adjusted to 2.5 mm. Tumor growth was assessed starting at day 7 after implantation with bioluminescence imaging (IVIS Spectrum, PerkinElmer; Waltham, MA, USA). For the treatment group, Auranofin (5 or 10 mg/kg/day) or PBS was administered to mice by oral gavage starting at day 7 after implantation. Mice were sacrificed when they began to show symptoms of continuous discomfort. Brains were collected and fixed in 4% formaldehyde for HE staining and IHC analysis.

### Statistical analysis

The relationship between gene expression levels was determined using Pearson correlation analysis. Kaplan-Meier survival curves were generated and compared using the log-rank test. The two-tailed χ^2^ test was used to analyze the association between *NONO* expression and clinicopathological characteristics. Paired or unpaired Student's t-tests for two-group comparison and one-way analysis of variance (ANOVA) for multi-group comparisons were performed using GraphPad Prism 8.0 (La Jolla, CA, USA). Data for each group were represented as the mean standard error of the mean (SEM) and *P* values < 0.05 were considered to be statistically significant.

### Data Availability

The datasets analyzed during the current study are available in the TCGA and the CGGA websites. The data generated in this study are publicly available in Gene Expression Omnibus (GEO) at GSE190950 and GSE191021.

## Results

### The splicing factor NONO is overexpressed in GBM

**Figure 1:**
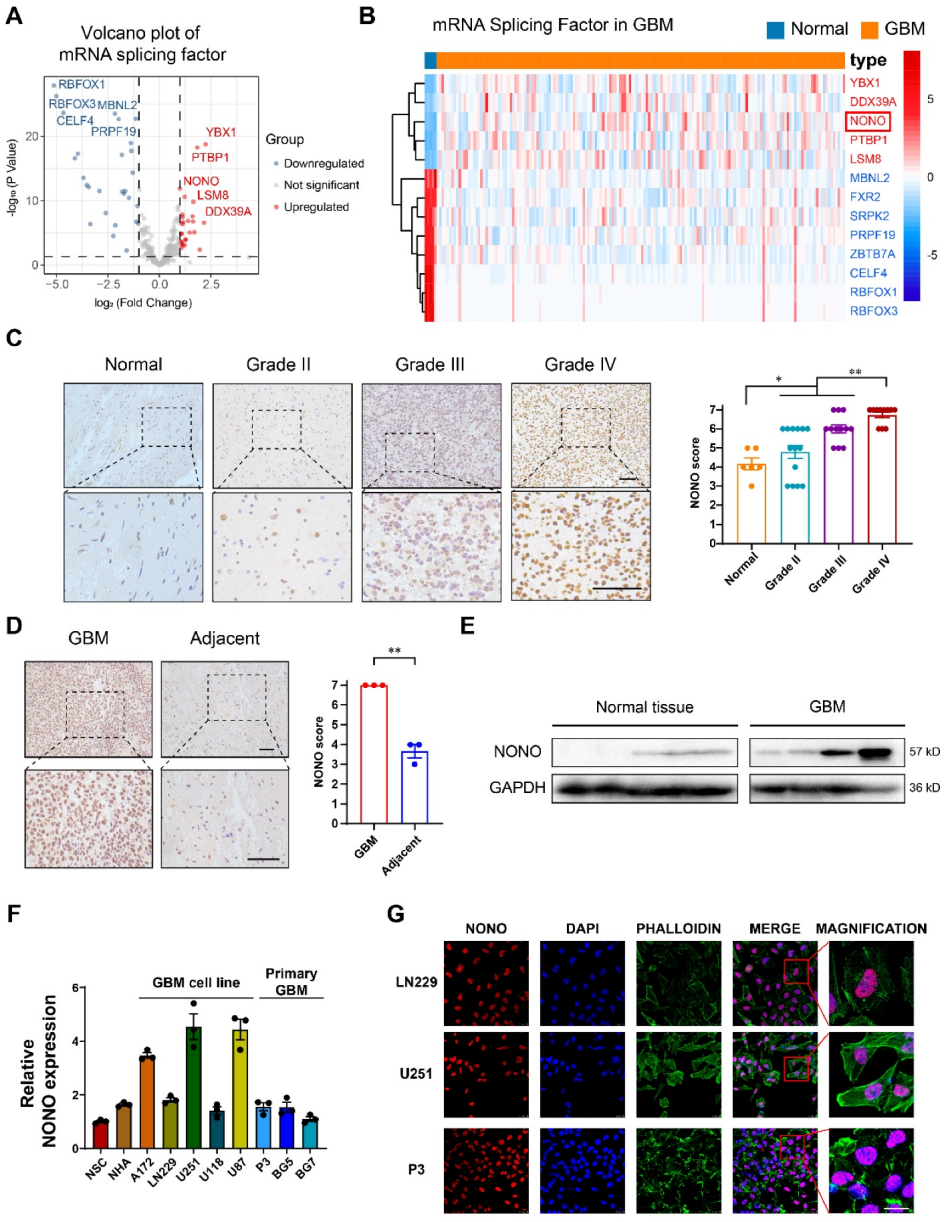
** Expression levels of splicing factor NONO are upregulated in glioma and associated with tumor grade. A** Volcano plot showing the fold-change (log2) in expression levels of mRNA-splicing-related genes (n = 355) based on GBM (n = 168) vs non-neoplastic brain tissue samples (n = 5). Data were obtained from the TCGA dataset. **B** The expression heatmap of the differentially expressed mRNA-splicing-related genes between GBMs and non-neoplastic brain tissue samples from the TCGA dataset. **C** Representative images of IHC staining of NONO in non-neoplastic brain (n = 6) and different pathological grades of gliomas (n = 37), and scoring. Scale bar = 100 μm. **D** Representative images of NONO IHC staining in GBM and adjacent brain tissues from 3 paired samples, and scoring. Scale bar = 100 μm. **E** Western blot analysis of NONO expression in non-neoplastic brain tissue and GBM samples (n = 3). **F** qRT-PCR analysis of *NONO* mRNA expression in 2 non-cancer cell lines and 8 GBM cell lines. *GAPDH* was used for normalization (n = 3).** G** Immunofluorescence staining showing NONO (red) subcellular localization (n = 3). Nuclei are stained with DAPI (blue) and actin filaments with Phalloidin (green). Scale bar = 25 μm. Data are shown as mean ± SEM. *P < 0.05, **P < 0.01.

To investigate the spliceosome as a potential target in GBM, we first identified 355 proteins with mRNA splicing function according to Gene Ontology. Further analysis was performed on the proteins associated with the term “mRNA splicing” (Table S2). Expression data of these splicing factors in GBM was obtained from the publicly available TCGA database, normalized and log2 transformed. EdgeR analysis demonstrated that the splicing factor *NONO* was significantly overexpressed in GBM (n = 168) compared with normal brain tissue (n = 5) (Figure 1A-B). To confirm the splicing potential of NONO, we performed GSEA using the CGGA dataset, and demonstrated that increased levels of *NONO* were associated with increased RNA splicing and mRNA processing (Figure S1A). Increased expression of *NONO* in GBM was also detected in the publicly available expression data obtained from the CGGA (Figure S1B). The increased expression of *NONO* in GBM was also correlated with expression changes in several other factors. *NONO* was positively correlated with epigenetic modifier enzymes *EZH2* and *HDAC2* and splicing factors *YBX1* and *DDX39A* in GBM from the CGGA (Figure S1C). Kaplan-Meier analysis of the top 3 significantly overexpressed splicing factors demonstrated that while *YBX1* and *PTBP1* did not show prognosis differences (Figure S1D), high expressing *NONO* tumors (LGG and GBM) exhibited shorter overall survival (OS) compared with low expressing *NONO* tumors based on the CGGA data (Figure S1E). Univariate and multivariate COX analysis of *NONO* illustrated that expression of *NONO* was an independent prognostic indicator in glioma (Figure S1F).

IHC staining of NONO performed on non-neoplastic brain (n = 6 cases) and an independent cohort of primary tumors (n = 37 cases) from Qilu Hospital demonstrated that protein levels of NONO were also significantly increased in GBM and correlated with increasing WHO tumor grade (Figure 1C). Stronger staining of NONO was also present in GBM tissues compared to the adjacent non-neoplastic tissue (Figure 1D). Western blot analysis performed in parallel on lysates prepared from the cohort of tumors confirmed the increase in NONO in GBM relative to non-neoplastic tissues (~ 6-fold; Figure 1E).

We also examined cell populations *in vitro*, including normal cell lines (NSC and NHA), GBM cell lines (A172, LN229, U251, U118 and U87) and patient-derived primary GSCs (P3, BG5 and BG7), for the transcription levels of *NONO* with RT-PCR. U251, U87, P3, and BG5 displayed higher levels of *NONO* (Figure 1F). Among these cell lines, U251 (high levels of *NONO*), LN229 (low levels of *NONO*) and P3 (primary GBM cells) were selected for further investigation. Immunofluorescence staining revealed nuclear localization of NONO which was consistent with its role as a splicing factor (Figure 1G).

In summary, the combined analysis demonstrated that overexpression of the splicing factor NONO predicted a worse prognosis in GBM.

### NONO knockdown inhibits GBM growth *in vitro* and *in vivo*

**Figure 2:**
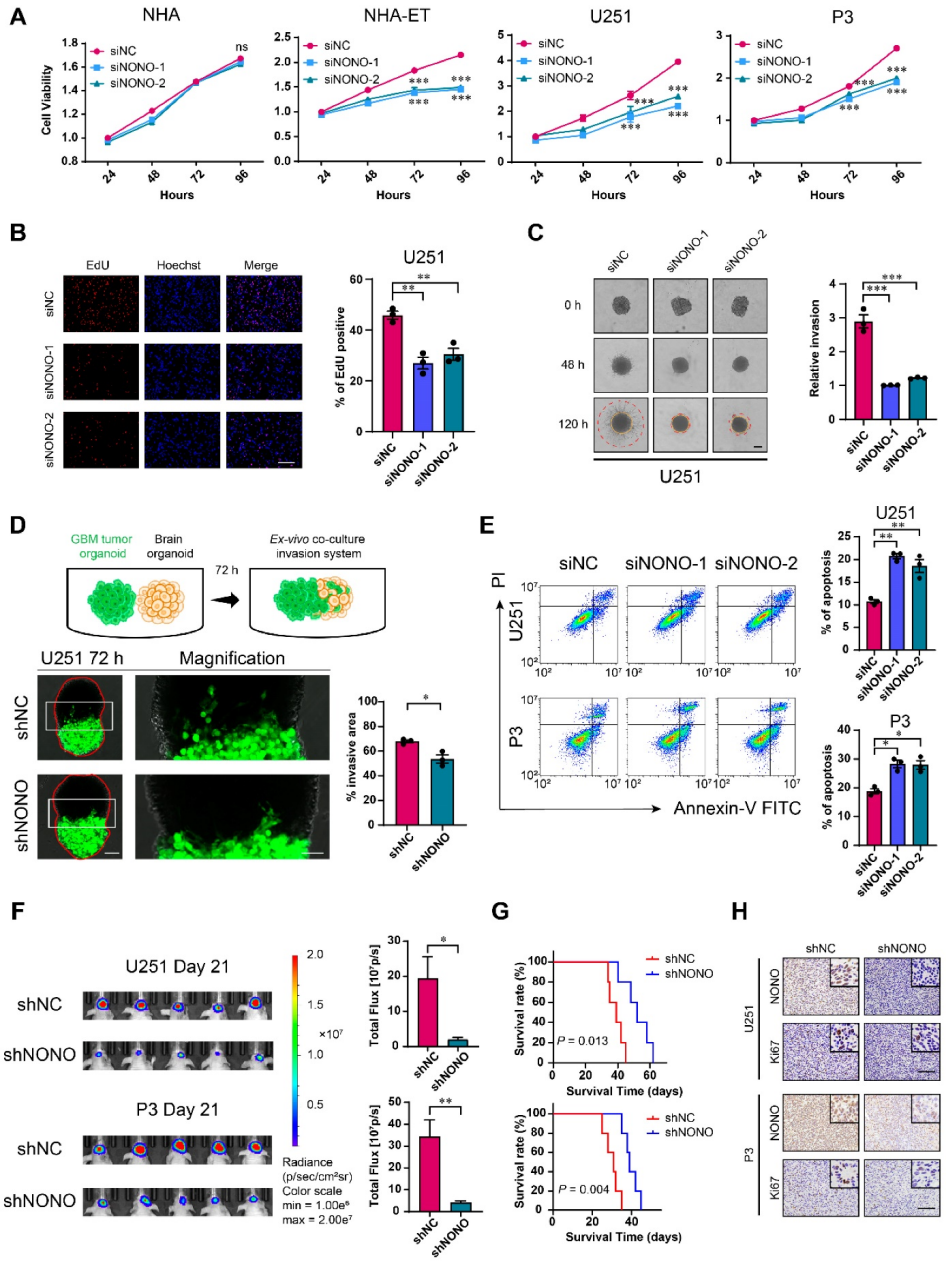
** Knockdown of NONO suppresses proliferation and invasion, and promotes apoptosis in GBM cell lines. A** CCK-8 assay for cell viability of NHA, NHA-ET, U251 and P3 after transfection with siNC or two different siRNAs (siNONO-1 and siNONO-2) (n = 3). Obtained data were normalized to the siNC group. **B** EdU assay to assess the cell growth of U251 transfected with siNC, siNONO-1 and siNONO-2 (n = 3). Scale bar = 100 μm. **C** 3D tumor spheroid invasion assay to measure invasion of U251 transfected with siNC, siNONO-1 and siNONO-2 (n = 3). Scale bar = 200 μm. **D** Model and representative images of co-culture invasion assays for U251 infected with lentiviral constructs expressing shNC or shNONO (n = 3). The invasion ability was evaluated at 72 h. Scale bar = 200 μm and 100 μm (magnified inset). **E** Flow cytometry analysis of Annexin V-FITC and propidium iodide (PI) staining for the detection of apoptosis in U251 and P3 transfected with siNC, siNONO-1 and siNONO-2 (n = 3). **F** Bioluminescence images of mice (n = 5 per group) orthotopically implanted with luciferase expressing U251 and P3 cells infected with lentiviral constructs expressing shNC or shNONO. Bioluminescence was collected to assess tumor growth. **G** Kaplan-Meier survival curve of groups of xenograft bearing mice (n = 5 per group). The Log-rank test was used to obtain statistical significance.** H** IHC for NONO and Ki67 levels in GBM xenografts. Scale bar = 100 μm. Data are shown as mean ± SEM. *P < 0.05, **P < 0.01, ***P < 0.001.

To determine the potential role of NONO in the development of GBM, we knocked down NONO in different cell lines with 2 siRNAs (Figure S2A-C). GBM cell lines and GSCs transfected with siRNAs against NONO exhibited reduced cell viability (Figure 2A and Figure S2D). In contrast, knockdown of NONO did not alter cell viability of normal NHA. Loss of NONO also led to reduced proliferation of U251 and P3 cells in the EdU assay (~ 10%; Figure 2B and Figure S2E).

We next generated cell populations (U251 and P3) stably expressing shRNAs (shNC and shNONO) through lentiviral infection (Figure S2F) and examined cell proliferation. The number of colonies formed was reduced in U251- and P3-shNONO cells relative to controls (Figure S2G). Cell cycle parameters assessed with flow cytometry also revealed an increase in the percentage of U251- and P3-shNONO cells in S phase. This result was consistent with previous experiments 22. Thus, inhibition of proliferation with loss of NONO may be partly mediated by cell cycle arrest in S phase (Figure S2H).

We also examined the invasive properties of GBM cell lines transfected with siRNAs against NONO in transwell migration and 3D spheroid invasion assays. The number of cells crossing the membrane was decreased (~ 40%) as was the relative invasion of spheres in the 3D assay relative to control cell populations (Figure 2C and Figure S3A-B). To more closely imitate the physiologically invasive tumor microenvironment, we also established a novel co-culture invasion model of tumor spheroids with normal rat brain organoids as previously described 20. In this ex-vivo model, the tumor spheres from U251- and P3-shNONO cells exhibited less invasive ability into the rat brain organoids compared with control cell populations (Figure 2D and Figure S3C).

We next used flow cytometry to determine whether U251 and P3 transfected with siRNAs against NONO were undergoing apoptosis. Cells transfected with siNONO exhibited increased staining with Annexin-V and PI, markers of apoptosis (Figure 2E). Western blot analysis demonstrated that loss of NONO in U251 and P3 cells reduced levels of proteins involved in the epithelial-mesenchymal transition (EMT) but increased those involved in apoptosis, thus confirming its role in the promotion of tumor growth (Figure S3D). RT-PCR of EMT markers and cell cycle checkpoint molecules also demonstrated that knockdown of NONO caused loss of EMT core and S/G2 phase related proteins (Figure S3E).

Sphere forming ability was suppressed in BG5 and BG7 GSCs transfected with siRNAs against NONO (Figure S3F-H). Thus, the inhibition of NONO also suppressed the self-renewal of GSCs.

To explore the loss of NONO on GBM cells *in vivo*, U251- and P3-shNONO and shNC (control) cells were injected into mouse brains (4-week-old nude mice; n = 5 for each group) to generate orthotopic xenografts. Growth of orthotopic xenografts derived from U251- and P3-shNONO cells was significantly reduced, and overall survival of tumor-bearing mice was prolonged (52 days vs 39 days, and 40 days vs 30 days, U251-shNONO and P3-shNONO vs U251-shNC and P3-shNC, respectively; Figure 2F-G and Figure S3I). IHC staining for the proliferation marker Ki-67 was decreased in tumors derived from U251-and P3-shNONO cells, which was consistent with suppressed tumor growth (Figure 2H).

Taken together, the inhibition of NONO suppressed glioma proliferation *in vitro* and *in vivo*, and promoted apoptosis. Loss of NONO furthermore inhibited invasion and self-renewal properties typical of GBM.

### NONO promotes GBM progression *in vitro* and *in vivo*

**Figure 3:**
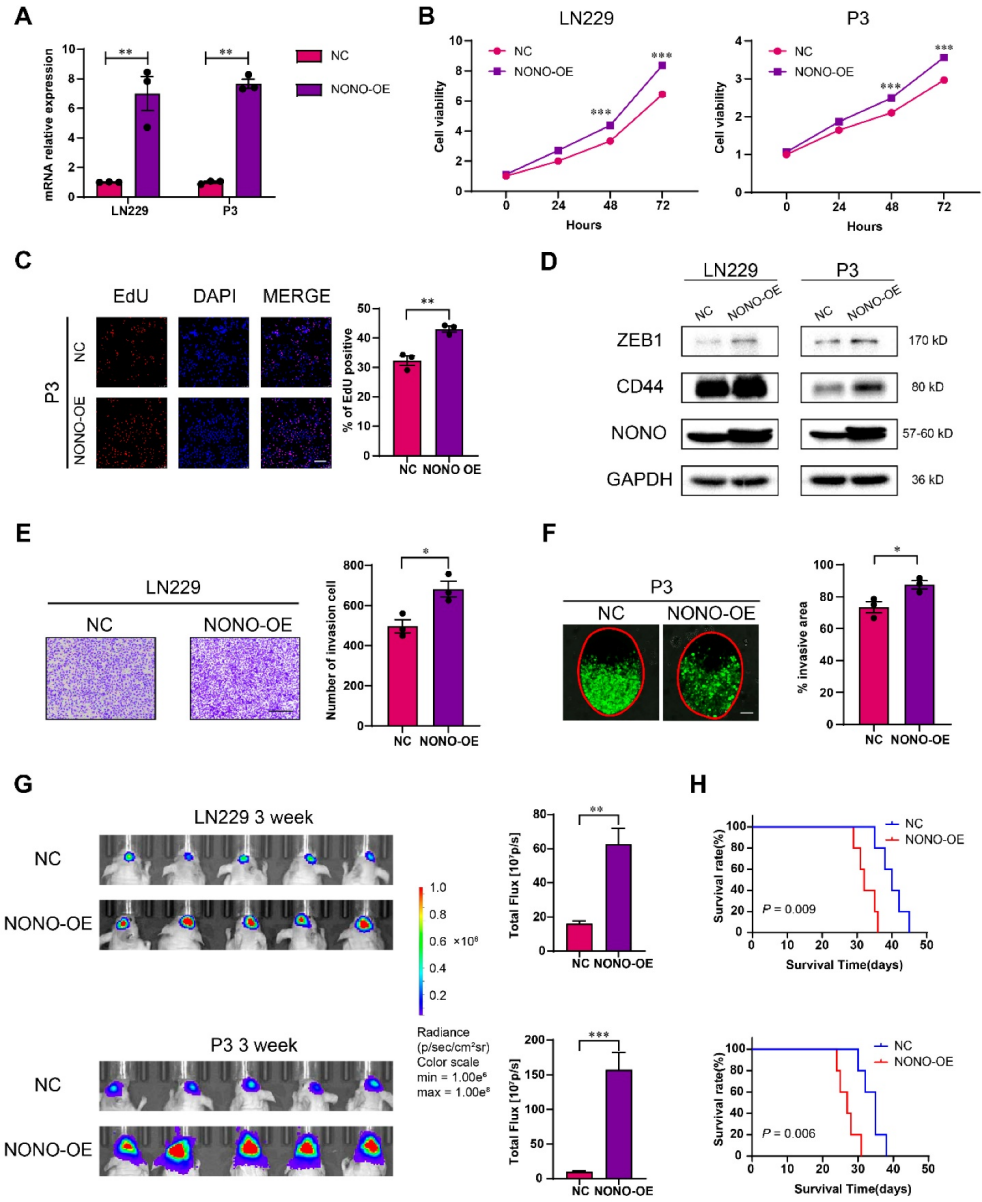
** Overexpression of NONO promotes the proliferation and invasion of GBM. A** *NONO* mRNA expression levels in LN229 and P3 after infection with lentivirus expressing FLAG-NONO (NONO-OE) or control sequence (NC) (n = 3). *GAPDH* was used for normalization. **B** CCK-8 assay to determine cell viability of LN229- and P3-NONO-OE or NC. Obtained data were normalized with the NC group (n = 3). **C** EdU assay to assess the cell growth of P3-NONO-OE or -NC (n = 3). Scale bar = 100 μm. **D** Western blot analysis for EMT related proteins and NONO in LN229- and P3-NONO-OE or NC (n = 3). **E** Transwell assay to evaluate invasion of LN229-NONO-OE or -NC (n = 3). Scale bar = 100 μm. **F** *Ex vivo* co-culture invasion assays for P3-NONO-OE or -NC (n = 3). The invasion ability was evaluated at 72 h. Scale bar = 200 μm. **G** Bioluminescence images of mice (n = 5 per group) orthotopically implanted with luciferase expressing LN229- and P3-NONO-OE or -NC. The bioluminescence was collected for assessment of tumor growth. **H** Kaplan-Meier survival curve of corresponding groups of mice (n = 5 per group). Log-rank test was used to obtain statistical significance. Data are shown as mean ± SEM. *P < 0.05, **P < 0.01, ***P < 0.001.

We next infected LN229 and P3 cells with lentiviral expression constructs for NONO (Figure 3A). CCK8, EdU and colony forming assays demonstrated that overexpression of NONO enhanced cell viability and proliferation (Figure 3B-C and Figure S4A-B). NONO also induced ZEB1 and CD44 in LN229 and P3 cells, and promoted their migration and invasion properties in transwell and *ex vivo* invasion assays (Figure 3D-F). These results were consistent with the results from GSEA indicating an association between EMT and NONO-high tumors (Figure S4C). In addition, xenografts derived from LN229- and P3-NONO-OE showed enhanced growth relative to controls and significantly shortened OS of mice (Figure 3G-H and Figure S4D).

Taken together, the overexpression of NONO promoted growth and invasion of LN229 and P3 cells *in vitro* and promoted growth of GBM *in vivo*.

### Loss of NONO induces intron retention of *GPX1* and *CCN1* and NONO binds the intron of pre-mRNAs

**Figure 4:**
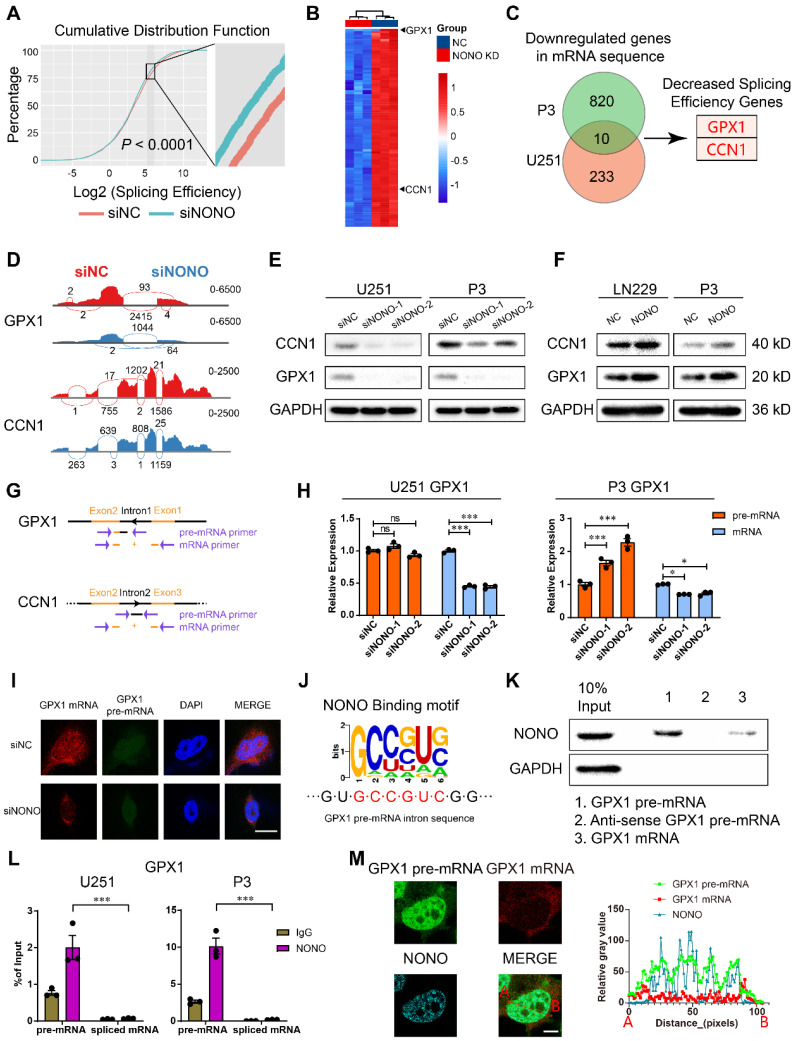
** Expression of *GPX1* and *CCN1* is regulated by NONO-mediated pre-mRNA splicing. A** Global splicing efficiency analysis at splicing sites after transfection of U251 and P3 cells with siNC and siNONO. Splicing efficiency was determined as “transread count/5' and 3' intron end first base coverage”.** B** The top 100 downregulated genes in the P3 sequencing data based on siNC versus siNONO. The expression data was z-transformed. **C** Venn plot displaying the significantly downregulated mRNAs in both U251 and P3 sequencing data. The genes with decreased splicing efficiency were selected based on splicing analysis.** D** Sashimi plot visualization of RNA-seq reads mapping to *GPX1* and *CCN1* in U251 cells in response to NONO knockdown. **E** Western blot analysis for GPX1 and CCN1 of U251 and P3 transfected with siNC and siNONO (n = 3). **F** Western blot analysis for GPX1 and CCN1 of LN229- and P3-NONO-OE or -NC (n = 3). **G** The schematic representation of primers designed for pre-mRNA and mRNA of *GPX1* and *CCN1*. **H** qRT-PCR analysis of pre-mRNA and mRNA for *GPX1* in U251 and P3 transfected with siNC, siNONO-1 and siNONO-2 (n = 3). *GAPDH* was used for normalization.** I** RNA FISH probes for pre-mRNA or mRNA were used for detection in U251 (n = 3). Scale bar = 20 μm.** J** The NONO binding motif in the intron of *GPX1* pre-mRNA. **K** RNA pulldown assay with *GPX1* pre-mRNA, mRNA and anti-sense pre-mRNA to detect binding with NONO in U251 (n = 3).** L** The RIP-PCR assay with NONO to detect *GPX1* pre-mRNA and mRNA (n = 3). Input was used for normalization and IgG was used for negative control.** M** Representative images of RNA FISH for *GPX1* pre-mRNA (green) and GPX1 mRNA (red), and immunofluorescence for NONO (blue) in U251 (n = 3). The right diagram shows the relative gray value of staining on the X-axis (AB). Scale bar = 25 μm. Data are shown as mean ± SEM. *P < 0.05, **P < 0.01, ***P < 0.001.

To explore the mechanism of NONO involved in the development of GBM, we performed high throughput RNA-seq of U251 and P3 cells transfected with siNONO and the control, siNC. The global splicing efficiency of intron-exon junctions was significantly downregulated in U251 and P3 cells transfected with siNONO relative to controls (Figure 4A). The expression of the EMT related genes *CD44* and *ZEB1* was also downregulated in the siNONO group (Figure S5A). The global landscape of mapped introns was also consistent with an increase in intron retention in cells with loss of NONO (Figure S5B). Previous articles have identified SC35 as an important splicing factor in the nucleus 23, and associated the morphological form of immunofluorescent SC35 dots with splicing potential 24. The subcellular distribution of the nuclear speckle marker SC35 was altered, with more collapsed and decreased SC35 dots in the nuclei of cells with NONO knockdown (Figure S5C).

We identified downregulated genes in both cell lines and found 10 genes in common. We performed further analysis on *GPX1* and *CCN1* based on their expression levels in the GBM expression data from the TCGA database and the decreased splicing efficiency (Figure 4B-C and Table S3-4). Sashimi plot visualization of *GPX1* and *CCN1* revealed suppression of these mRNAs, and that the sequencing peak of their introns did not decrease, but instead, increased or remained stable (Figure 4D and Figure S5D). These results indicated that NONO mediated the intron splicing of *GPX1* and *CCN1*. The expression of GPX1 and CCN1 was also examined at the protein level (Figure 4E). While loss of NONO led to reduced GPX1 and CCN1, the overexpression of NONO upregulated protein levels of GPX1 and CCN1 (Figure 4F).

To directly assess intron retention in *GPX1* and *CCN1* genes, we performed RT-PCR and qPCR with primers specific for an intron-exon junction in the pre-mRNA and an exon junction in the mature mRNA (Figure 4G). Although the levels of the mRNAs were significantly decreased, the levels of the pre-mRNAs remained constant or slightly increased (Figure 4H and Figure S5E-F). The increase of mRNA but not pre-mRNA was also observed in the NONO-OE group (Figure S5G-H). In addition to intron retention, we explored other splicing types of NONO (Figure S5I). The results indicated that NONO not only influenced intron retention but also skipped exon, alternative 3′ splice site, alternative 5′ splice site and mutually exclusive exons. While taking into consideration the entire splicing efficiency (Figure 4A), we focused on intron retention after NONO knockdown. The decrease in *GPX1* mRNA was also detected with FISH, which furthermore demonstrated that the distribution of pre-mRNA was still mainly localized to the nucleus (Figure 4I and Figure S6A).

The function of NONO as a mediator of intron splicing has been reported previously 16. To confirm a splicing function for NONO in GBM, we first verified the binding between NONO and *GPX1* or *CCN1* pre-mRNA. Considering the function of NONO as an RNA binding protein and the published viewpoint that NONO is mainly found bound to introns, we looked for NONO binding motifs in the introns of *GPX1* (Figure 4J) and *CCN1* of pre-mRNAs (Figure S6B). We found consensus binding sequences in the *GPX1* pre-mRNA and examined the binding between the pre-mRNA and NONO through RNA pull-down analysis. NONO was pulled down with the pre-mRNA rather than the spliced form or the anti-sense control (Figure 4K and Figure S6C). We then examined the binding efficiency of NONO with *GPX1* and *CCN1* pre-mRNAs and mature mRNAs, using RIP-PCR (Figure 4L and Figure S6D). The results demonstrated that NONO preferentially bound to pre-mRNAs rather than mature mRNAs. In addition, the colocalization of *GPX1* pre-mRNA rather than mRNA with NONO was observed in the nucleus (Figure 4M).

In summary, NONO mediated the splicing of *GPX1* and *CCN1* by directly binding to introns in the pre-mRNAs. Though the high expression of *GPX1* and *CCN1* both indicated worse prognosis in glioma, only *GPX1* was significantly upregulated in GBM compared with normal brain tissue (Figure S6E-F). Therefore, we focused further study on the function of GPX1 downstream of NONO. We plotted the PSI (percent spliced in) percentage of intron retention of *GPX1* against the expression data of NONO in glioma from the TCGA dataset to examine the splicing relationship between the two genes (Figure S6G). The r value of -0.22 demonstrated that the NONO level was negatively correlated with the level of *GPX1* intron retention. The ENCODE project ENCSR861PAR, eCLIP experiment in K562 for NONO also revealed the binding of NONO to *GPX1* introns (Figure S6H). Furthermore, knockdown of GPX1 did not influence protein levels of NONO (Figure S6I), and overexpression or loss of NONO did not affect the transcription of *GPX1* and *CCN1* (Figure S6J).

### NONO inhibition impairs redox homeostasis through the expression of GPX1

**Figure 5:**
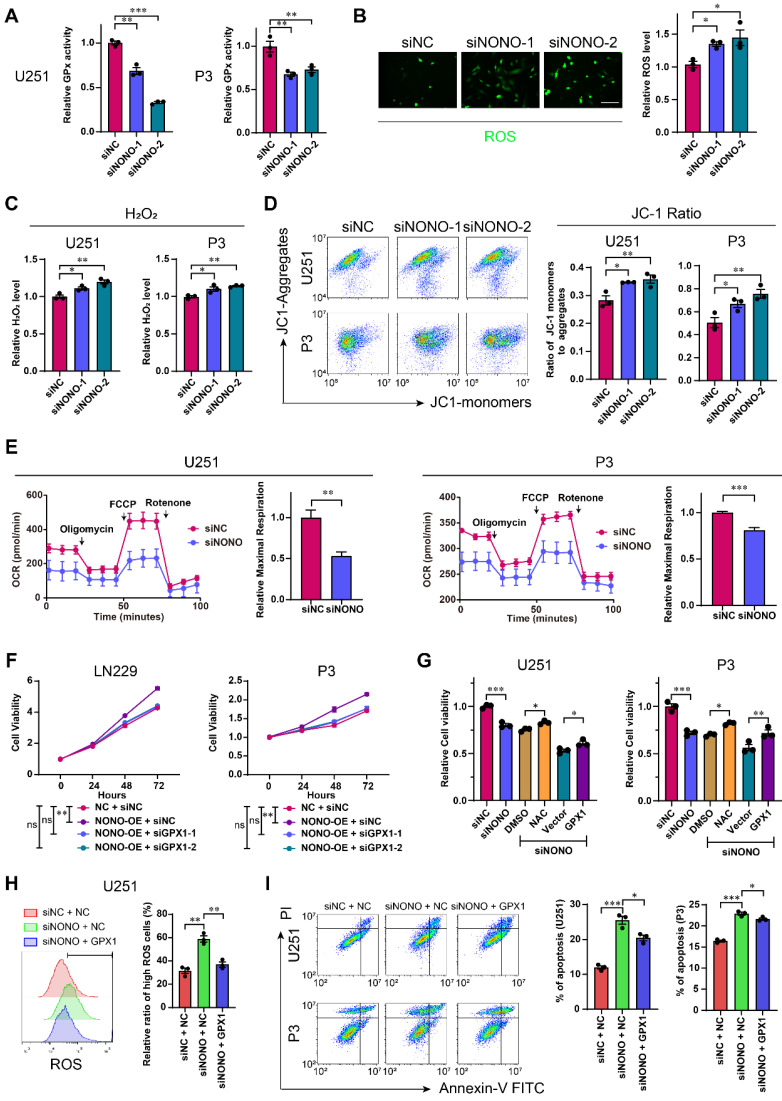
** Loss of NONO induces redox imbalance through the GPX1 pathway. A** GPx assay for U251 and P3 transfected with siNC, siNONO-1 and siNONO-2 (n = 3). GPx ability of cells transfected with siNC was used for normalization. **B** Reactive Oxygen Species Assay for U251 transfected with siNC, siNONO-1 and siNONO-2 (n = 3). ROS levels in cells transfected with siNC were used for normalization. Scale bar = 100 μm. **C** Hydrogen Peroxide Assay for U251 and P3 transfected with siNC, siNONO-1 and siNONO-2 (n = 3). The H_2_O_2_ level in cells transfected with siNC was used for normalization. **D** Flow cytometric detection of JC-1 staining of U251 and P3 transfected with siNC, siNONO-1 and siNONO-2 (n = 3). FL2 corresponds to oxidized JC1 (JC1-Aggregate) and FL1 corresponds to non-oxidized JC1 (JC1 Monomers). **E** Seahorse assay for detection of the maximal respiration rate for U251 and P3 transfected with siNC and siNONO (n = 3). Maximal respiration rate of cells transfected with siNC was used for normalization. **F** CCK-8 assay for relative cell viability for rescue experiments in LN229- and P3-NONO-OE or -NC transfected with siNC, siGPX1-1 and siGPX1-2 (n = 3). Cell viability of “NC + siNC” was used for normalization.** G** CCK-8 assay for relative cell viability for rescue experiments as determined at 48 h in U251 and P3 (n = 3). Cell viability of cells transfected with siNC was used for normalization.** H** Flow cytometric detection of ROS for U251-NC or -GPX1 transfected with siNC or siNONO (n = 3). The bar graph shows the ratio of cells with ROS levels higher than the given threshold.** I** Flow cytometry to detect Annexin V-FITC and PI staining to assess apoptosis U251- and P3-NC or -GPX1 transfected with siNC or siNONO (n = 3). Data are shown as mean ± SEM. *P < 0.05, **P < 0.01, ***P < 0.001.

GPX1 is one of the most important antioxidant enzymes in humans, and catalyzes the reaction of hydrogen peroxide with glutathione to maintain redox homeostasis. GPX1 is thought to be responsible for most GPX activity 25. To investigate the potential role of the NONO-GPX1 axis in the development of GBM, we performed GO enrichment analysis based on the RNA-seq data. In addition to invasion and proliferation related functions, the knockdown of NONO was associated with the induction of the response to reactive oxygen species (Figure S7A). We first determined GPX activity using the cellular glutathione peroxidase assay. The results demonstrated that GPX activity was significantly inhibited after knockdown of NONO in U251 and P3 cells (Figure 5A). In addition, the suppression of NONO increased ROS levels, including H_2_O_2_, and downregulated the levels of GSH, which indicated an imbalance in redox homeostasis (Figure 5B-C and Figure S7B). As excess hydrogen peroxide can affect mitochondria, we examined the mitochondrial membrane potential (Δψ). SiRNA transfected cells were exposed to the JC1 probe and fluorescence of the probe was detected with flow cytometry. The increase in JC1+ cells (the ratio of green/red fluorescence) revealed a reduced mitochondrial activity compared to the control group (Figure 5D). In addition, the maximal oxygen consumption rate (OCR) as determined in the seahorse assay, was impaired in cells transfected with siNONO relative to controls (Figure 5E).

We then examined whether the impact of NONO on GBM was through the regulation of GPX1. We first demonstrated the efficiency of GPX1 siRNAs and the expression construct in U251 and P3 cells (Figure S7C). The loss of GPX1 led to reduced cell viability and promoted apoptosis in both U251 and P3 cells (Figure S7D-E). The decrease in GPX1 also led to reduced expression of EMT related genes and 3D invasion in both cell lines (Figure S7F-G).

We also performed a rescue experiment and observed that the enhanced cell viability induced by NONO overexpression was blocked through loss of GPX1 (Figure 5F and Figure S8A). Moreover, the reduced viability of U251 or P3 cells transfected with siNONO was partially rescued with either the H_2_O_2_ inhibitor NAC, which did not promote cell viability alone (Figure S8C), or overexpression of GPX1 (Figure 5G and Figure S8B). The overexpression of GPX1 in cells with NONO knockdown also recovered the increased levels of ROS and apoptosis (Figure 5H-I and Figure S8D). Collectively, the recovery of GPX1 protein levels partly relieved the imbalance of redox homeostasis and the increase in apoptosis. Finally, the reduced invasion induced by loss of NONO was also rescued by GPX1 (Figure S8E-F).

In summary, knockdown of NONO in GBM cell lines was partially rescued through overexpression of GPX1.

### The RNA-recognition motifs 2 (RRM2) domain of NONO binds introns and requires PSPC1

**Figure 6:**
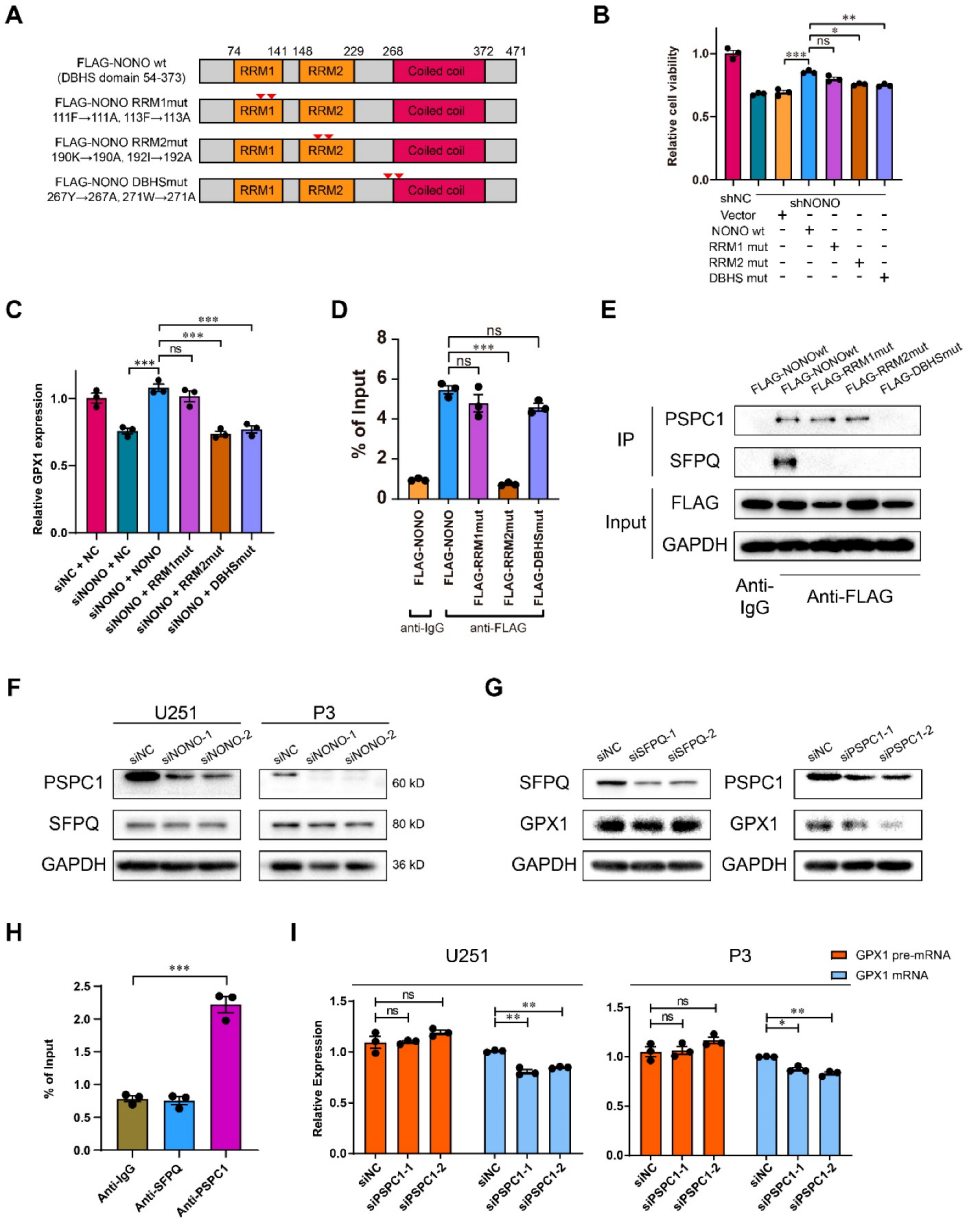
** RRM2 domain of NONO binds *GPX1* pre-mRNA and requires PSPC1 for splicing. A** Schematic diagram of NONO illustrating the positions of *NONO* mutations.** B** CCK-8 assay for relative cell viability of mutant rescue experiments as determined at 48 h in U251 (n = 3). Cell viability of U251-shNC was used for normalization.** C** Relative expression of *GPX1* mRNA in U251 cells transfected with siNC, siNONO and rescued by NONO mutations (n = 3). **D** The RIP-PCR assay of FLAG-NONOwt or 3 FLAG-NONOmut for *GPX1* pre-mRNA (n = 3). Input was used for normalization, and IgG was used for the negative control. **E** CoIP and subsequent western blot showing the interaction between NONOwt or 3 NONOmut with PSPC1 or SFPQ (n = 3).** F** Western blot to detect expression of PSPC1 and SFPQ in U251 and P3 transfected with siNC, siNONO-1 and siNONO-2 (n = 3). **G** Western blot to detect GPX1 in U251 transfected with siNC, siSFPQ-1 and siSFPQ-2, or siPSPC1-1 and siPSPC1-2 (n = 3).** H** The RIP-PCR assay to detect SFPQ or PSPC1 binding with *GPX1* pre-mRNA (n = 3). Input was used for normalization and IgG was used for the negative control.** I** qRT-PCR analysis of *GPX1* pre-mRNA and mRNA in U251 and P3 transfected with siNC, siPSPC1-1 and siPSPC1-2 (n = 3). *GAPDH* was used for normalization. Data are shown as mean ± SEM. *P < 0.05, **P < 0.01, ***P < 0.001.

To investigate the domain required for NONO-mediated GPX1 expression, we generated U251 cells stably expressing FLAG-tagged wildtype NONO and a series of constructs mutated at two sites (Figure 6A). The sites of mutation were designed based on previous work 16, 26. Only the RRM1mut restored cell viability to the same levels as wildtype NONO, indicating that the RRM2 and DBHS domains were necessary for NONO function (Figure 6B). In addition, only the overexpression of RRM1mut rescued the expression of *GPX1* mRNA (Figure 6C). As the DBHS domain is considered to be the binding site of the heterodimers of the DBHS protein family, we examined whether the RRM2 domain mediated the binding to RNA. RIP-PCR of these mutants confirmed that the RRM2 domain was required for NONO to bind the pre-mRNA (Figure 6D).

To further investigate the inability of DBHSmut to rescue cell viability and whether NONO formed heterodimers with other DBHS family members (Figure S8G), we assessed the relationship of SFPQ and PSPC1 to NONO in GBM. The correlation between SFPQ and NONO was weaker than for PSPC1 and NONO (Figure S8H). Using CoIP assays, we found that wildtype NONO interacted with both PSPC1 and SFPQ, but that the DBHSmut bound neither of them, and that the RRM1mut and the RRM2mut showed stronger binding to PSPC1 (Figure 6E). In addition, the protein levels of PSPC1 decreased with knockdown of NONO. However, the levels of SFPQ remained constant (Figure 6F).

The expression of GPX1 mediated by NONO was also reduced with knockdown of PSPC1, but not SFPQ (Figure 6G). To further investigate the interaction between NONO and DBHS family members, we examined whether PSPC1 or SFPQ bound to *GPX1* pre-mRNA. PSPC1 but not SFPQ bound to the pre-mRNA. Furthermore, knockdown of PSPC1 also inhibited the splicing of *GPX1* (Figure 6H-I). PSPC1 was not upregulated in LGG and GBM in the TCGA dataset (Figure S8I), although the knockdown of PSPC1 impaired the binding of NONO to pre-mRNA (Figure S8J). Loss of PSPC1 also did not affect the expression of NONO (Figure S8K). These experiments confirmed that PSPC1 interacted with NONO and was required for NONO-mediated splicing of *GPX1*.

### Auranofin interferes with the NONO-GPX1 axis and is a potential molecular agent for the treatment of GBM

**Figure 7:**
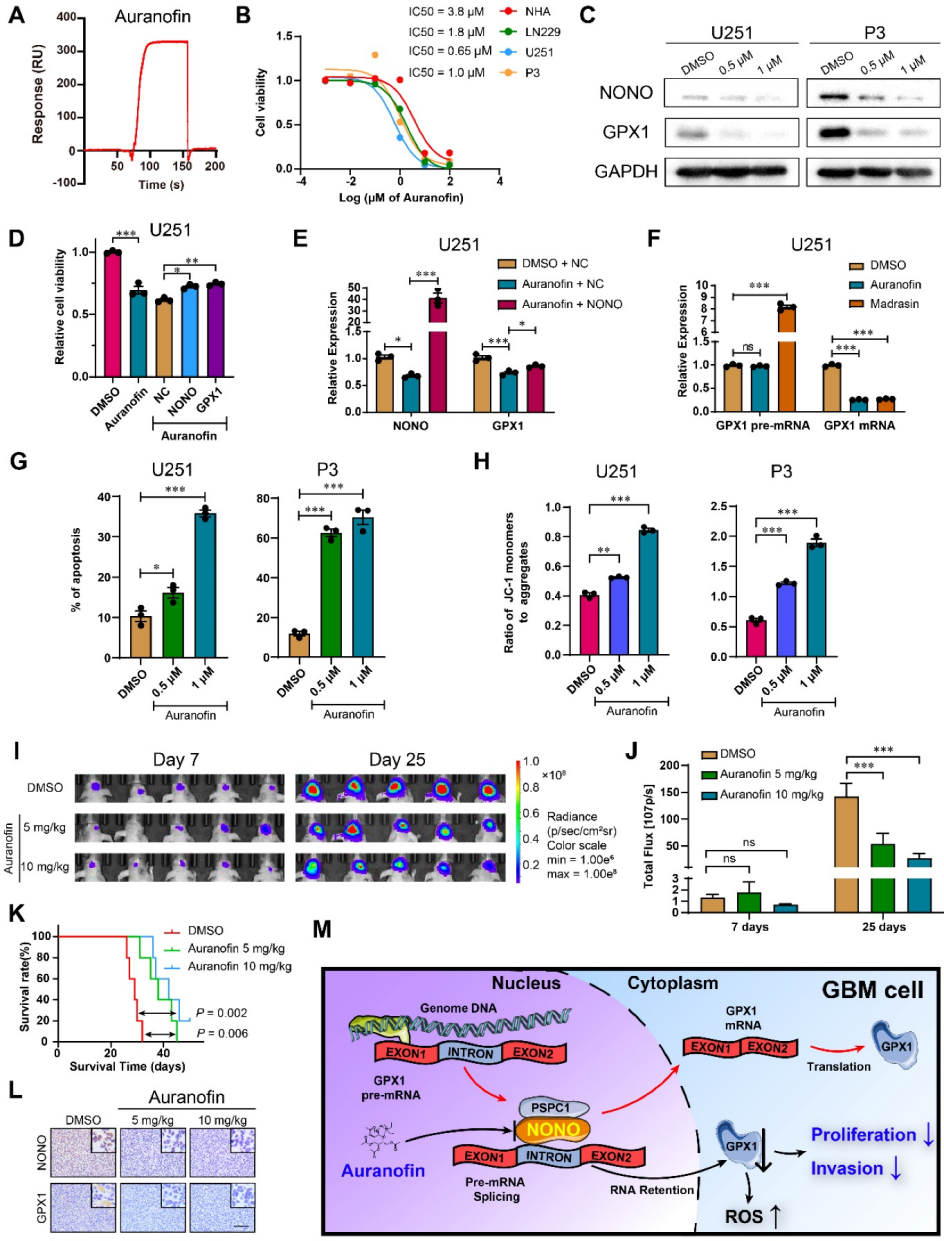
** Auranofin inhibits GBM through the NONO-GPX1 pathway. A** Red lines represent the global interaction of Auranofin with NONO in the SPR assay (n = 3). **B** CCK-8 assay for relative cell viability of NHA, LN229, U251 and P3 treated with Auranofin (n = 3). Dose-inhibition curve and calculation of IC50s.** C** Western blot to detect NONO and PSPC1 in U251 and P3 treated with DMSO, 0.5 μM or 1 μM Auranofin (n = 3). **D** CCK-8 assay for relative cell viability of Auranofin-treated U251 rescue experiments with NONO and GPX1 as determined at 48 h (n = 3). Cell viability of the DMSO group was used for normalization. **E** qRT-PCR analysis of *NONO* and *GPX1* mRNAs in U251-NC or -NONO after 1 μM Auranofin treatment (n = 3). *GAPDH* was used for normalization. **F** qRT-PCR analysis of *GPX1* pre-mRNA and mRNA in U251 after DMSO, 1 μM Auranofin or 30 μM Madrasin treatment (n = 3). *GAPDH* was used for normalization.** G** Statistics of apoptosis for U251 and P3 treated with DMSO, 0.5 μM or 1 μM of Auranofin (n = 3).** H** Statistics of JC1-Aggregate and JC1 Monomers of U251 and P3 treated with DMSO, 0.5 μM or 1 μM of Auranofin (n = 3). **I, J** Bioluminescence images at day 7 and day 25 for mice (n = 5 per group) orthotopically implanted with luciferase expressing P3 cells treated with DMSO, 5 mg/kg or 10 mg/kg Auranofin. The bioluminescence was collected for analysis of tumor growth.** K** Kaplan-Meier survival curve of groups of xenograft bearing mice (n = 5 per group). The Log-rank test was used to obtain statistical significance.** L** IHC for NONO and GPX1 levels in the GBM xenografts. Scale bar = 100 μm. **M** Schematic diagram of Auranofin targeting NONO-mediated splicing of *GPX1* pre-mRNA. Data are shown as mean ± SEM. *P < 0.05, **P < 0.01, ***P < 0.001.

We then screened for potential drugs targeting NONO. An FDA approved drug for rheumatoid arthritis, Auranofin, which has anti-cancer activity 27, 28, was also found to inhibit GPX1 in GBM 29. However, the mechanism underlying this inhibition was not determined. We therefore performed *in vitro* SPR assays, which demonstrated considerable affinities and direct binding between Auranofin and NONO (Figure 7A). In cells in culture, the half maximal inhibitory concentration (IC50) of Auranofin for NHA was higher than for GBM cell lines, indicating possible specificity of Auranofin for tumor cells (Figure 7B). We subsequently chose the concentrations of 0.5 μM and 1 μM for functional assays. The protein levels of NONO and GPX1 were both decreased in U251 and P3 cells treated with Auranofin (Figure 7C), and the overexpression of either NONO or GPX1 partly rescued the decrease in cell viability caused by Auranofin (Figure 7D and Figure S9A). Auranofin also inhibited the mRNA levels of *NONO* and *GPX1*. However, overexpression of NONO in Auranofin-treated cells rescued *GPX1* mRNA expression levels (Figure 7E and Figure S9B). Still, Auranofin may also downregulate NONO mRNA and protein through additional different mechanisms as previously reported 30.

To determine the mechanism of Auranofin-induced inhibition of GPX1, we first examined the inhibition efficiency of Auranofin on *GPX1* mRNA under different levels of NONO. The percentage of inhibition for *GPX1* mRNA in cells under Auranofin treatment increased along with increasing NONO levels, indicating that the loss of *GPX1* mRNA was mediated by NONO (Figure S9C). Proliferation, as assessed in the EdU assay, was also rescued by GPX1 overexpression in cells under Auranofin treatment (Figure S9D). We then compared *GPX1* mRNA levels in cells under treatment with Auranofin or the global splicing inhibitor Madrasin 31. Both drugs downregulated the mature mRNA levels of *GPX1*. However, although the levels of *GPX1* pre-mRNA did not increase in cells under Auranofin treatment to the levels under Madrasin treatment, the pre-mRNA levels remained constant as for NONO knockdown (Figure 7F and Figure S9E). Auranofin also promoted apoptosis and caused a decrease in mitochondrial function (Figure 7G-H and Figure S9F-G). Moreover, Auranofin inhibited invasion of GBM cells in transwell and 3D invasion assays (Figure S9H-I).

Based on the *in vitro* experiments, we assessed the anti-glioma efficiency of Auranofin *in vivo*. P3 orthotopic xenografts were established in nude mice, which were then randomized into 3 groups: Auranofin at either 5 or 10 mg/kg/2d; or DMSO (vehicle control). Auranofin significantly inhibited tumor growth and prolonged OS of tumor bearing animals (40 days vs 28 days, treated and untreated animals, respectively; Figure 7I-K and Figure S9J). IHC staining of xenograft tissues revealed decreased expression of NONO and GPX1 (Figure 7L), as well as the proliferation marker Ki-67 (Figure S9K).

Auranofin inhibited global splicing through multiple mechanisms, including disturbing SC35 agglomerates (Figure S10A), preventing NONO binding to pre-mRNA (Figure S10B) and promoting the degradation of NONO protein (Figure S10C), indicating that Auranofin functioned as a splicing inhibitor. However, Auranofin did not alter the localization of NONO protein (Figure S10D). *In vivo*, the knockdown and overexpression of NONO also reduced or promoted the expression of *GPX1* mRNA, respectively, while the level of *GPX1* pre-mRNA remained unaltered (Figure S10E-F). Auranofin treatment *in vivo* also led to reduced expression of *GPX1* mRNA although pre-mRNA remained unchanged as expected (Figure S10G).

Taken together, Auranofin inhibited GBM progression *in vivo*, possibly through targeting NONO-mediated *GPX1* splicing. Thus, Auranofin might have potential as a repurposed drug for the treatment of GBM patients.

## Discussion

In humans, NONO is involved in the normal processing of pri-miRNA and pre-mRNA 32. However, NONO has also been associated with the development of disease, such as cancer initiation and progression, by altering the splicing of specific transcripts. In hepatic carcinoma for example, NONO was shown to promote tumorigenesis by causing a switch in the alternative splicing of *BIN1* 33. In this study, we first identified 355 proteins with mRNA splicing function according to Gene Ontology. Among these proteins, NONO was further examined and determined to be abnormally upregulated in GBM through analysis of TCGA and CGGA datasets. We then found that NONO contributed to the malignancy of GBM by promoting proliferation and invasion. We furthermore explored splicing mechanisms and observed intron retention in specific genes due to the loss of NONO. Using RIP-PCR and pulldown assays, we demonstrated that NONO bound to introns in specific pre-mRNAs, *GPX1* and *CCN1*, and that NONO bound these pre-mRNAs through its RRM2 domain. The loss of NONO altered the splicing of *GPX1* and led to inhibition of proliferation and invasion of GBM. Finally, we demonstrated that the small molecule inhibitor Auranofin blocked NONO activity and inhibited GBM tumor growth in an *in vivo* orthotopic xenograft model. Thus, NONO might serve as a therapeutic target in the treatment of GBM.

Alternative pre-mRNA splicing is an evolutionarily conserved post-transcriptional process 34, which is classified into five types of events: skipped exon, alternative 5' splice site, alternative 3' splice site, mutually exclusive exons and intron retention 35. Aberrant regulation of alternative splicing is a molecular feature of human cancers 36. Intron retention has been shown to be a widespread mechanism that contributes to tumor-suppressor inactivation through analysis of the RNA sequencing data from a large number of cancer patient samples 37. However, while skipped exon is considered to be the most common mechanism leading to alternative splicing in cancer, intron retention has thus far been largely underestimated 38. According to the results made *in vitro* and *in vivo* in our study, we identified NONO as an overexpressed splicing factor and demonstrated that NONO knockdown induced intron retention, which inhibited tumor growth in GBM. The mechanism underlying intron retention in GBM was thus further investigated.

We identified two specific splicing targets regulated by NONO based on the RNA-seq data, *GPX1* and *CCN1*. Intron retention in *CCN1* pre-mRNA was previously reported 39, 40. However, based on GBM data from the TCGA, *CCN1* levels showed equivalent transcription levels between GBM tumor and non-neoplastic brain tissue samples. However, we found GPX1 to be significantly upregulated in GBM compared to normal brain. Therefore, we focused on the function of GPX1. GPX1 is a selenocysteine-containing peroxidase enzyme that protects mammalian cells from oxidative stress, especially the endogenous ROS molecule H_2_O_2_ 25. The fact that GPX1 is an enzyme raises the possibility that an inhibitor of GPX1 could become a candidate molecule for the treatment of GBM. However, due to the shallow active site of GPX1, only a few inhibitors have been identified to date 41. Our results demonstrated that redox imbalance and apoptosis induced in cells are partially mediated through the loss of NONO and subsequent intron retention of the *GPX1* pre-mRNA.

NONO, SFPQ and PSPC1 often carry out their function as heterodimers and have been identified as multifunctional molecules with specific roles in cellular processes dependent on different cellular contexts 9. For example, SFPQ was shown to mediate the coupling of NONO and targeting of exons in hepatic carcinoma 33. In GBM, we found PSPC1 to interact with NONO for the splicing of pre-mRNAs and showed greater correlation of expression with NONO than SFPQ. PSPC1, however, is not likely to bind introns directly due to the absence of the motif. Therefore, the exact function of PSPC1 in the NONO-mediated splicing complex needs further investigation. As a multifunctional protein, the role of NONO in the formation of paraspeckles 42, RNA transport 43 and epigenetic regulation 15 has been found to promote cancer progression. In addition, NONO has been recently shown to promote TAZ phase separation in GBM which drives the oncogenic transcriptional program 44. Our study provides a new mechanism for the contribution of NONO to the development of GBM. However, the function of NONO in cancer requires further investigation.

The small molecule inhibitor Auranofin has been proposed as an inhibitor of NONO in triple-negative breast cancer 17. Auranofin is an FDA approved anti-inflammatory drug used in rheumatoid arthritis. Yet, several studies have demonstrated that Auranofin exhibits anti-proliferative properties in various cancers and could be repurposed 45. In glioma, Auranofin induced mitochondrial suppression through the GPX1 pathway 29. For the first time, we showed the direct binding of Auranofin to NONO *in vitro* and demonstrated that Auranofin exerts an anti-cancer activity in GBM cells *in vitro* and *in vivo*. The treatment of GBM with Auranofin is currently under investigation in a clinical trial (ClinicalTrials.gov Identifier: NCT02770378). The phase I study is aiming to assess the “CUSP9v3 Treatment Protocol”, which is a combination therapy of nine FDA proved non-oncological drugs with temozolomide. Our work demonstrates that Auranofin suppresses GBM through targeting intron splicing. The mechanism of the combination therapy of temozolomide and Auranofin, and how Auranofin influences the splicing function of NONO, require further investigation.

In conclusion, our results have demonstrated that overexpression of the splicing factor NONO promotes GBM progression, and the inhibition of NONO leads to intron retention in *GPX1*. The decrease in GPX1 induced apoptosis and inhibition of invasion due to an increase in ROS. Moreover, Auranofin exhibited anti-cancer activity in GBM cells through targeting the NONO-GPX1 axis. These findings provide a novel therapeutic strategy to treat GBM patients (Figure 7M).

## Supplementary Material

Supplementary figures and tables.

## Abbreviations

GBM: glioblastoma multiforme
LGG: low-grade glioma
pre-mRNA: precursor messenger RNA
RBPs: RNA binding proteins
RRMs: RNA-recognition motifs
GSEA: Gene set enrichment analysis
TCGA: The Cancer Genome Atlas
CGGA: Chinese Glioma Genome Atlas
OS: Overall survival
IHC: Immunohistochemistry
ROS: Reactive oxygen species
GSH: Glutathione
FISH: Fluorescence *in situ* hybridization
RIP: RNA immunoprecipitation
IP: Immunoprecipitation
IC50: half maximal inhibitory concentration
CHX: Cycloheximide
