# Supplementary material for: Targeting the splicing factor NONO inhibits GBM progression through GPX1 intron retention
Source: Theranostics. 2022 Jul 11;12(12):5451–69. doi: 10.7150/thno.72248 (PMC9330516; doi:10.7150/thno.72248)
Supplement: Supplementary file 1 — Supplementary figures and tables. [file thnov12p5451s1.pdf]

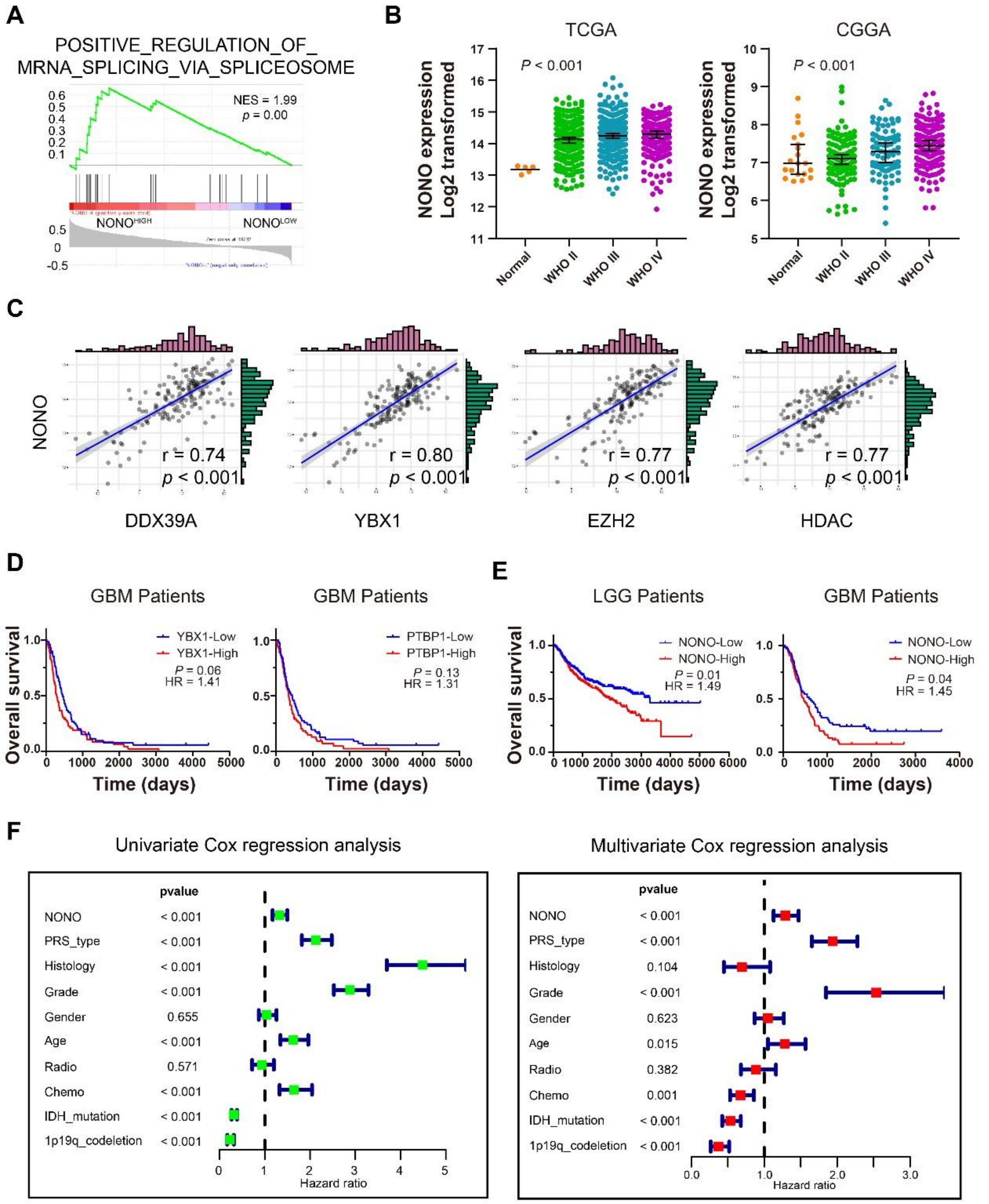

Figure S1. NONO is an upregulated mRNA splicing factor in glioma and is correlated with poor prognosis.

A GSEA enrichment showing the positive association of NONO-high coregulated genes with mRNA-Splicing-Via-Spliceosome gene signatures. The expression data was obtained from the CGGA. NES = normalized enrichment score. **B** *NONO* expression analysis in

non-neoplastic brain tissue and glioma based on TCGA (n = 675) and CGGA (n = 345) datasets. **C** The positive correlation between *DDX39A*, *YBX1*, *EZH2*, *HDAC* and *NONO* based on the CGGA GBM dataset (n = 174). **D** Kaplan–Meier analysis for patient OS based on *YBX1* and *PTBP1* expression in GBM from the CGGA dataset (n = 138). **E** Kaplan–Meier analysis for patient OS based on *NONO*-high versus *NONO*-low expression in LGG (n = 404) and GBM (n = 138). Data were obtained from the CGGA dataset and *P*-values were obtained from the log-rank test. **F** Univariate and multivariate Cox regression of *NONO* expression for overall survival in CGGA glioma patients.

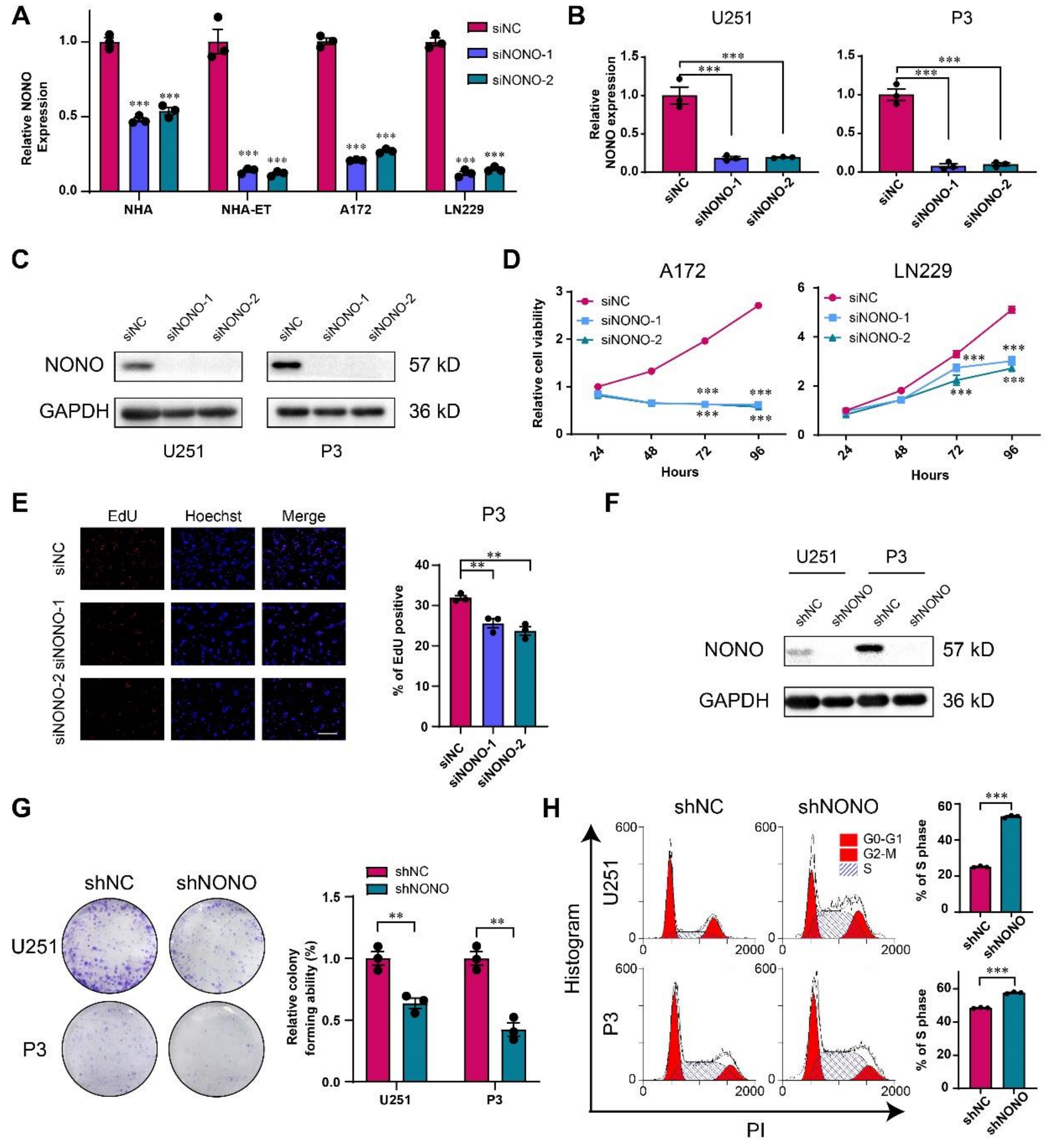

Figure S2. Knockdown of NONO suppresses cell growth in GBM.

A qRT-PCR analysis of *NONO* expression in NHA, NHA-ET and GBM cell lines transfected with siNC, siNONO-1 and siNONO-2 (n = 3). *GAPDH* was used for normalization. **B** Relative expression levels of *NONO* in U251 and P3 transfected with siNC, siNONO-1 and siNONO-2 (n = 3). *GAPDH* was used for normalization. **C** Western blot to detect NONO expression in U251 and P3 transfected with siNC, siNONO-1 and siNONO-2 (n = 3). **D** CCK-8 assay for cell viability of A172 and LN229 transfected with siNC, siNONO-1 and

siNONO-2 (n = 3). Obtained data were normalized with the siNC group. **E** EdU assay to assess the cell proliferation of P3 transfected with siNC, siNONO-1 and siNONO-2 (n = 3). Scale bar = 100 μm. **F** Western blot to detect NONO expression in U251- and P3-shNC or -shNONO (n = 3). **G** Colony forming assay for U251- and P3-shNC or -shNONO (n = 3). **H** Flow cytometry for cell cycle analysis of PI stained U251- and P3-shNC or -shNONO (n = 3). Data are shown as mean ± SEM. **P* < 0.05, ***P* < 0.01, ****P* < 0.001.

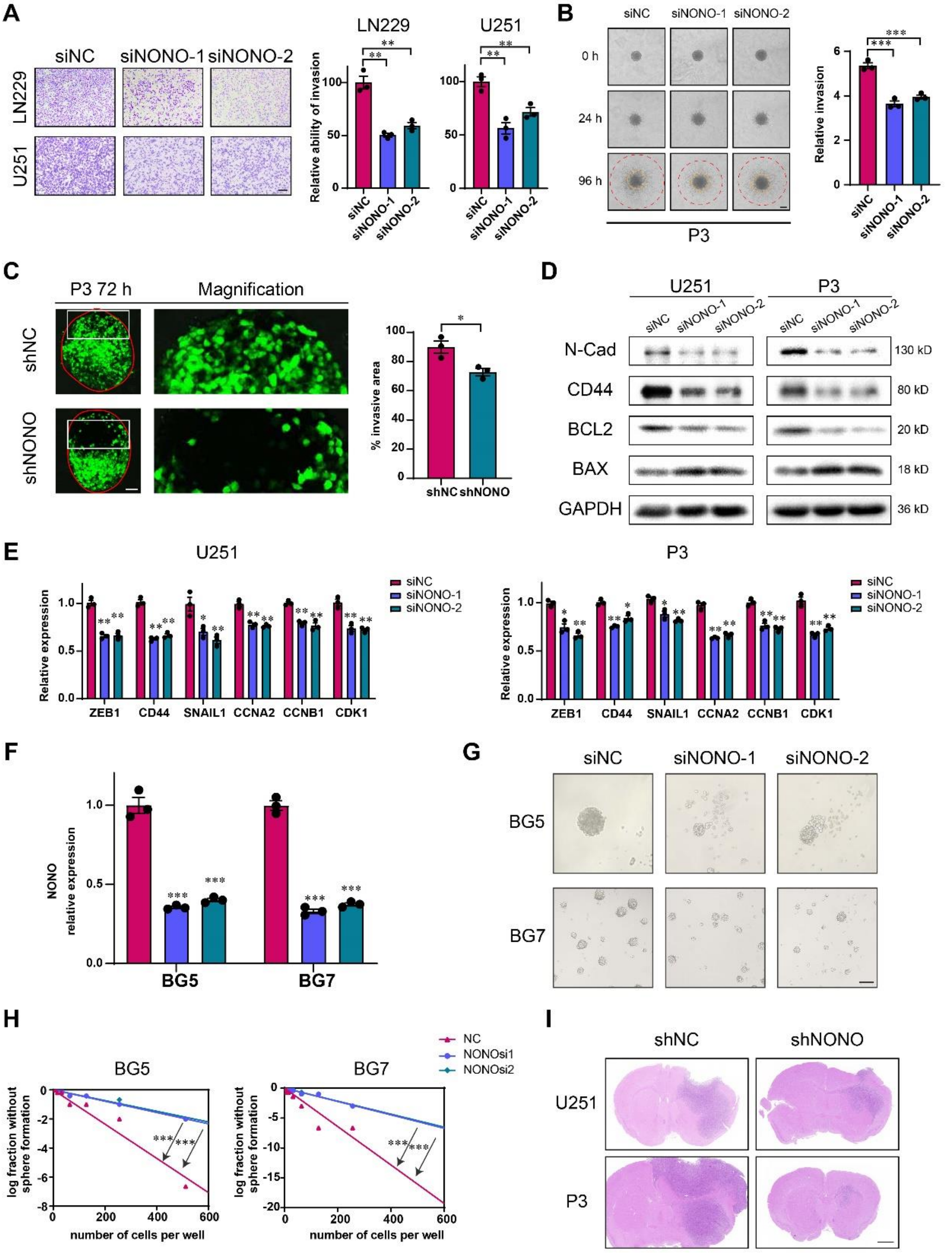

Figure S3. NONO knockdown inhibits invasion, sphere-forming ability and in vivo growth of GBM.

A Transwell assay of LN229 and U251 transfected with siNC, siNONO-1 and siNONO-2 (n = 3). Scale bar = 100 μm. **B** 3D tumor spheroid invasion assay to measure invasion of P3

transfected with siNC, siNONO-1 and siNONO-2 (n = 3). Scale bar = 200 μm. **C** Ex vivo co-culture invasion assays for P3-shNC or -shNONO (n = 3). The invasion ability was evaluated at 72 h. Scale bar = 200 μm. **D** Western blot to detect expression of N-cad, CD44, BCL-2 and BAX in U251 and P3 transfected with siNC, siNONO-1 and siNONO-2 (n = 3). **E** qRT-PCR analysis of expression of EMT related and cell cycle related genes in U251 and P3 transfected with siNC, siNONO-1 and siNONO-2 (n = 3). *GAPDH* was used for normalization. **F** The knockdown efficiency of NONO in GSCs BG5 and BG7 transfected with siNC, siNONO-1 and siNONO-2 (n = 3). **G** Representative images of tumorsphere formation assays for BG5 and BG7 transfected with siNC, siNONO-1 and siNONO-2 (n = 3). Scale bar = 100 μm. **H** Extreme limiting dilution assay performed for GSCs BG5 and BG7 transfected with siNC, siNONO-1 and siNONO-2 (n = 3). **I** Representative images of HE staining of mouse brains implanted with U251- or P3-shNC or -shNONO. Scale bar = 1 mm. Data are shown as mean ± SEM. *$P < 0.05$, **$P < 0.01$, ***$P < 0.001$.

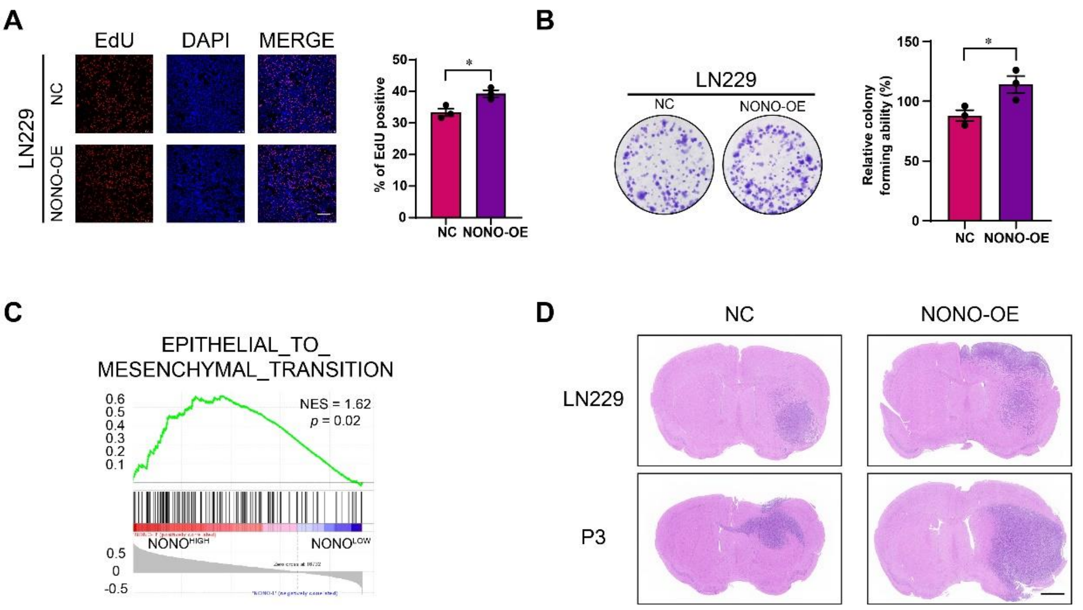

Figure S4. Overexpression of NONO promotes GBM proliferation in vitro and in vivo.

A EdU assay to assess the cell growth of LN229-NONO-OE or -NC (n = 3). Scale bar = 100 μm. **B** Colony forming assay for LN229-NONO-OE or -NC (n = 3). **C** GSEA enrichment showing the positive association of *NONO*-high coregulated genes with epithelial to mesenchymal transition gene signatures. The expression data was obtained from the CGGA. **D** Representative images of HE staining of mouse brains implanted with LN229- and P3-NONO-OE or -NC. Scale bar = 1 mm. Data are shown as mean ± SEM. $*P < 0.05$.

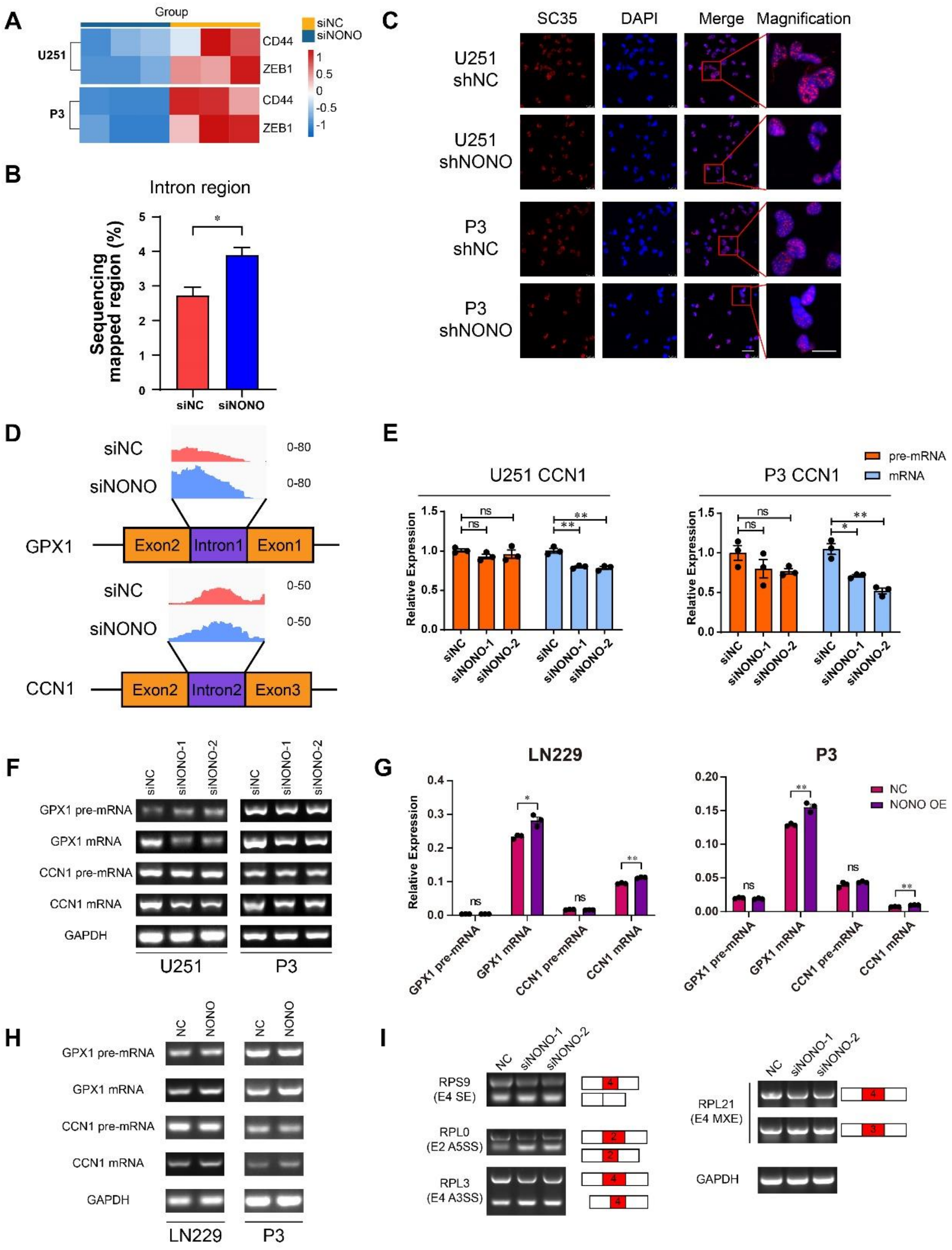

Figure S5. Expression of *GPX1* and *CCN1* is regulated by NONO-mediated pre-mRNA splicing.

A Heatmap displaying *CD44* and *ZEB1* expression obtained from RNA sequencing for U251 and P3 transfected with siNC and siNONO. **B** The percentage of reads of mRNA-

seq mapping to introns in the genome (n = 3). **C** Immunofluorescence images for SC35 (red) and nuclear (blue) staining for U251- and P3-shNC or -shNONO (n = 3). Scale bar = 50 μm and 25 μm (magnified inset). **D** The comparison of siNC and siNONO sequencing peaks in introns from U251. **E** qRT-PCR analysis of *GPX1* and *CCN1* mRNA expression in U251 and P3 transfected with siNC, siNONO-1 and siNONO-2 (n = 3). *GAPDH* was used for normalization. **F** Semi-quantitative PCR of pre-mRNA and mRNA for *GPX1* and *CCN1* in U251 and P3 transfected with siNC, siNONO-1 and siNONO-2 (n = 3). **G** qRT-PCR analysis of pre-mRNA and mRNA for *GPX1* and *CCN1* in LN229- and P3-NONO-OE or NC (n = 3). *GAPDH* was used for normalization. **H** Semi-quantitative PCR analysis of *GPX1* and *CCN1* pre-mRNA and mRNA in LN229- and P3-NONO-OE or NC (n = 3). *GAPDH* was used as a control. **I** Semi-quantitative PCR analysis for NONO-regulated AS events in 4 other representative genes in U251 cells (n = 3). *GAPDH* was used as a control. Data are shown as mean ± SEM. *$P < 0.05$, **$P < 0.01$, ***$P < 0.001$.

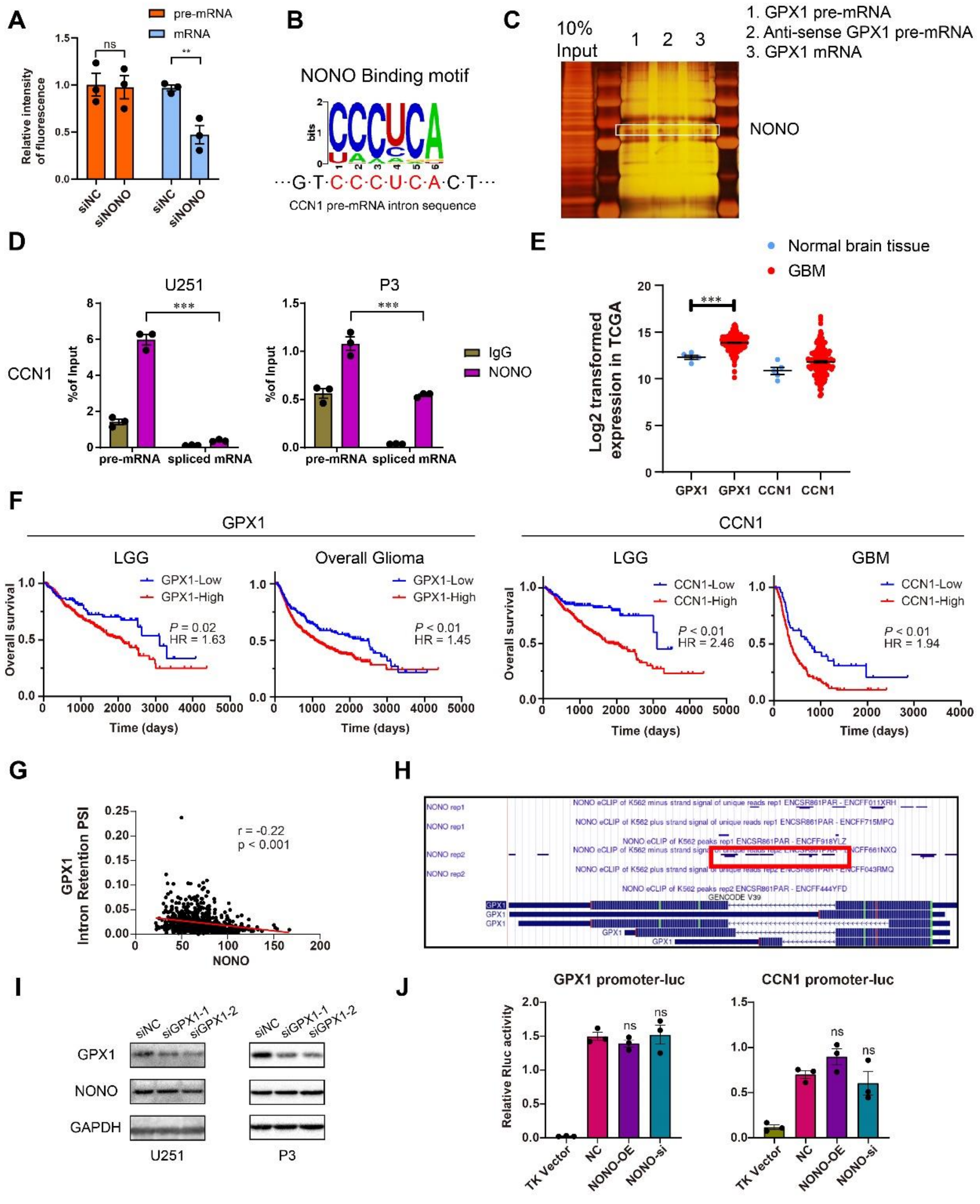

Figure S6. NONO binds the pre-mRNA of *GPX1* and *CCN1*, and *GPX1* is significantly upregulated in GBM.

A Quantification of FISH staining of *GPX1* pre-mRNA and mRNA in U251 cells (n = 3). **B** The NONO binding motif in the intron of the *CCN1* pre-mRNA. **C** Silver staining of SDS-PAGE to detect protein associated with RNA from a pulldown assay using *GPX1* pre-mRNA, mRNA and anti-sense pre-mRNA sequences (n = 3). **D** RIP-PCR assay for detection of

CCN1 pre-mRNA and mRNA binding with NONO (n = 3). Input was used for normalization and IgG was used for the negative control. **E** Log2 transformed expression data for *GPX1* and *CCN1* in GBM and non-neoplastic brain tissue samples from the TCGA database (Normal n = 5, GBM n = 169). **F** Kaplan–Meier analysis of patient OS based on *GPX1*-high versus *GPX1*-low expression in LGG (n = 409) and overall glioma (n = 624), and *CCN1*-high versus *CCN1*-low expression in LGG (n = 409) and GBM (n = 215). Data were obtained from the CGGA dataset and *P*-values were obtained from the log-rank test. **G** The correlation between *GPX1* PSI of intron retention and *NONO* expression in the TCGA dataset (n = 663). **H** The eCLIP project from ENCODE project ENCSR861PAR for NONO binding to *GPX1*. **I** Western blot to detect expression of NONO in U251 and P3 transfected with siNC, siGPX1-1 and siGPX1-2 (n = 3). **J** Luciferase activity detected for *GPX1* and *CCN1* promoter-dual-luciferase reporter gene assay constructs co-transfected into U251 cells with the NONO-OE construct or siNONO (n = 3). Data are shown as mean ± SEM. ***$P < 0.001$.

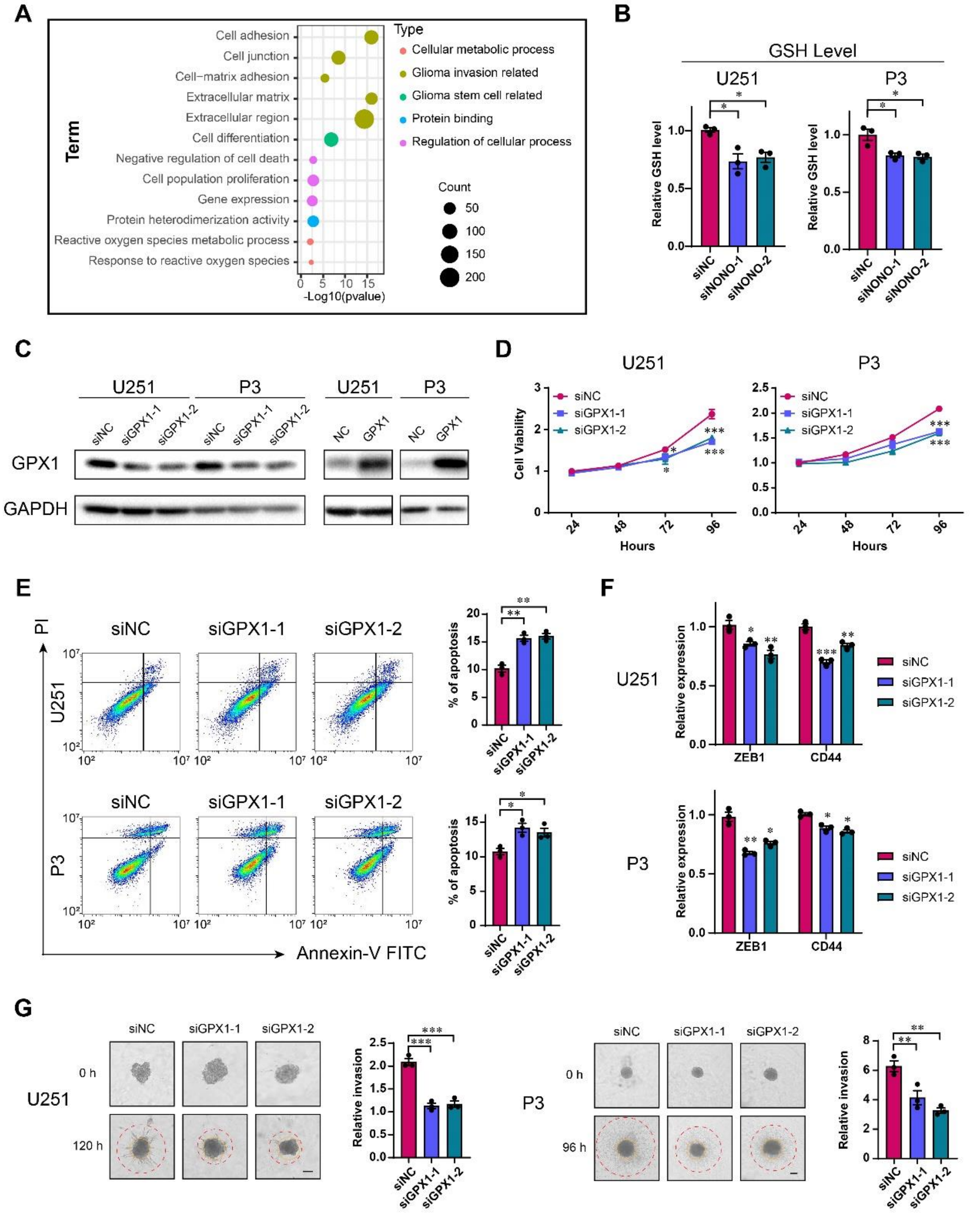

Figure S7. NONO inhibition impairs redox homeostasis and knockdown of GPX1 suppresses glioma proliferation and promotes apoptosis.

A GO enrichment of RNA-seq of U251 based on siNONO versus siNC. **B** Total glutathione assay for U251 and P3 transfected with siNC, siNONO-1 and siNONO-2 (n = 3). Total GSH levels for siNC were used for normalization. **C** Western blot analysis for siRNA knockdown

of GPX1 siNC, siGPX1-1 and siGPX1-2 and overexpression efficiency of plasmid expressing NC or GPX1 in U251 and P3 (n = 3). **D** CCK-8 assay for cell viability of U251 and P3 transfected with siNC, siGPX1-1 and siGPX1-2. Obtained data were normalized with the siNC group (n = 3). **E** Flow cytometry to detect Annexin V-FITC and PI staining to assess apoptosis in U251 and P3 transfected with siNC, siGPX1-1 and siGPX1-2 (n = 3). **F** qRT-PCR analysis of mRNA for EMT related genes *ZEB1* and *CD44* in U251 and P3 transfected with siNC, siGPX1-1 and siGPX1-2 (n = 3). *GAPDH* was used for normalization. **G** 3D tumor spheroid invasion assay for U251 and P3 transfected with siNC, siGPX1-1 and siGPX1-2 (n = 3). Scale bar = 200 μm. Data are shown as mean ± SEM. *$P < 0.05$, **$P < 0.01$, ***$P < 0.001$.

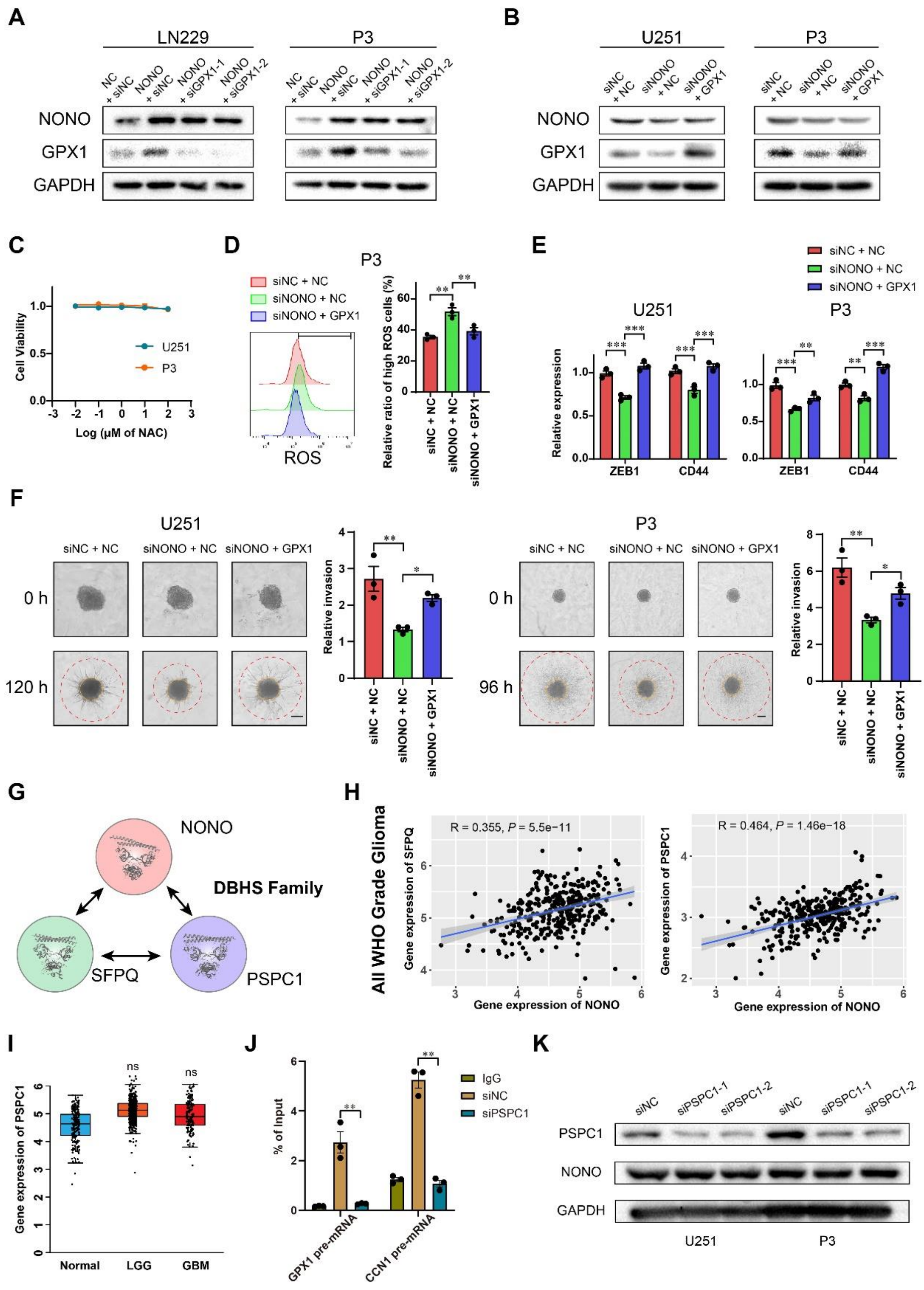

Figure S8. GPX1 rescues the knockdown of NONO and *NONO* is more highly correlated with *PSPC1*.

A Western blot analysis for GPX1 rescue in LN229 and P3 transfected with siNC, siGPX1-

1 or siGPX1-2 and plasmid expressing NC or NONO (n = 3). **B** Western blot analysis for GPX1 rescue in U251 and P3 transfected with plasmid expressing NC or NONO and siNC or siNONO (n = 3). **C** CCK-8 assay for cell viability of U251 and P3 after treatment with the H_2O_2 inhibitor NAC (n = 3). Obtained data were normalized with the 0.01 μM group. **D** Flow cytometric detection of ROS in P3 transfected with plasmid expressing NC or GPX1, and siNC or siNONO (n = 3). The bar graph shows the ratio of cells with ROS levels higher than the given threshold. **E** qRT-PCR analysis of mRNA for EMT related genes *ZEB1* and *CD44* in U251 and P3 transfected with plasmid expressing NC or GPX1 and siNC or siNONO (n = 3). *GAPDH* was used for normalization. **F** 3D tumor spheroid invasion assay for U251 and P3 transfected with plasmid expressing NC or GPX1 and siNC or siNONO (n = 3). Scale bar = 200 μm. **G** The relationship between proteins of the DBHS family. **H** Correlation of gene expression for *NONO* and *SFPQ* and *NONO* and *PSPC1* in gliomas from the CGGA (n = 325). **I** The expression levels of *PSPC1* in normal tissue (n = 207), LGG (n = 518) and GBM (n = 162) in TCGA and GTEx datasets. **J** The RIP-PCR assay with NONO to detect *GPX1* and *CCN1* pre-mRNA (n = 3). Input was used for normalization and IgG was used for the negative control. **K** Western blot to detect expression of NONO in U251 and P3 transfected with siNC, siPSPC-1 and siPSPC1-2 (n = 3). Data are shown as mean ± SEM. *$P < 0.05$, **$P < 0.01$, ***$P < 0.001$.

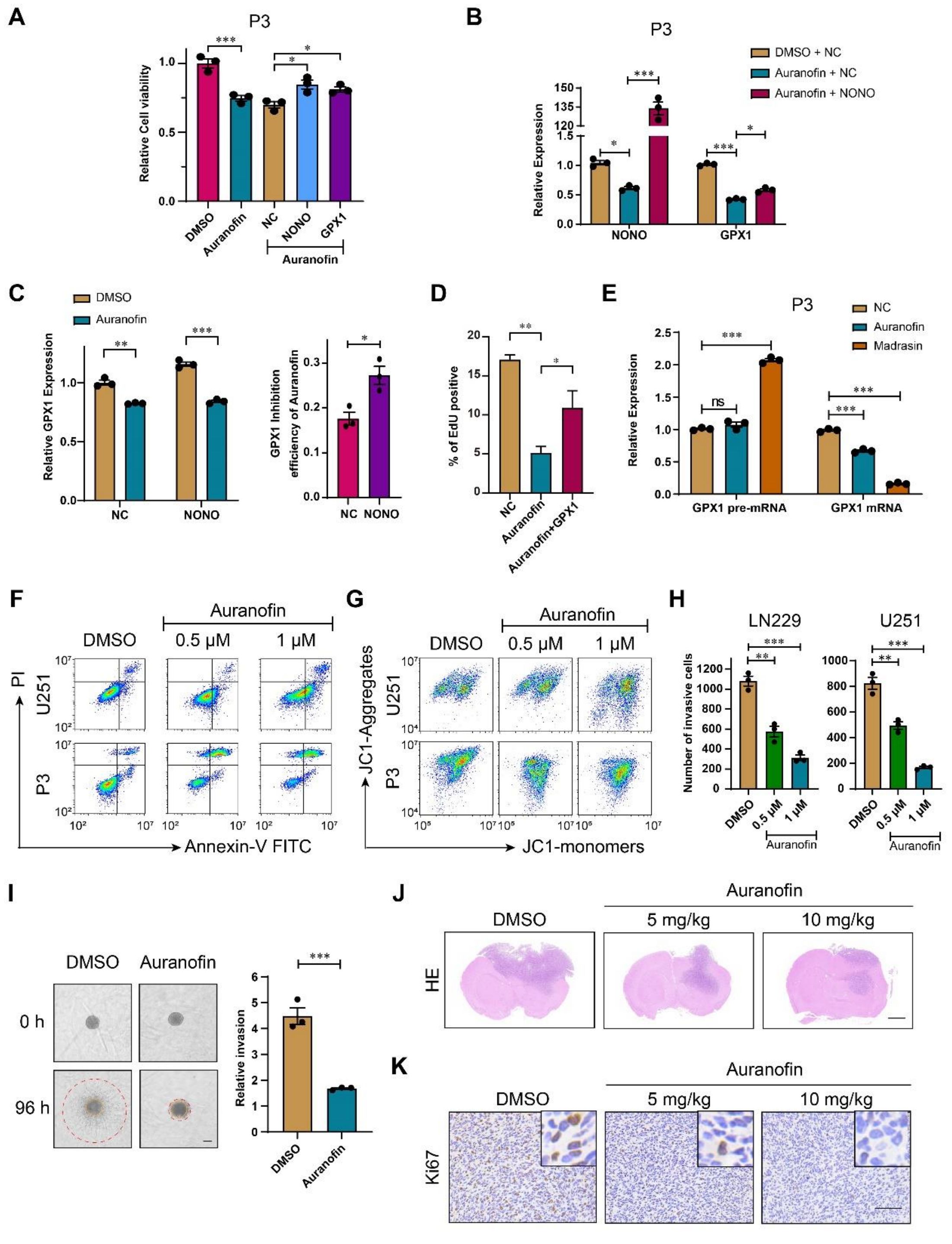

Figure S9. Auranofin inhibits NONO-mediated splicing, invasion and growth of glioma.

A CCK-8 assay for relative cell viability for Auranofin rescue experiments in P3 as determined at 48 h. Cell viability of the DMSO group was used for normalization (n = 3). **B**

qRT-PCR analysis of *NONO* and *GPX1* in P3 expressing NC or NONO and treated with 1 μM Auranofin (n = 3). *GAPDH* was used for normalization. **C** The expression of *GPX1* in parental U251 and U251-NONO-OE and treated with 1 μM Auranofin (n = 3). Relative inhibition efficiency of 1 μM Auranofin on *GPX1* mRNA in U251-NC and -NONO cells**. D** Statistical results for EdU assays for U251 cells after treatment with Auranofin or Auranofin and transfection with GPX1 cDNA (n = 3). **E** qRT-PCR analysis of *GPX1* pre-mRNA and mRNA in P3 treated with DMSO, 1 μM Auranofin or 30 μM Madrasin (n = 3). *GAPDH* was used for normalization. **F** Flow cytometry to detect Annexin V-FITC and PI staining to assess apoptosis in U251 and P3 treated with DMSO, 0.5 μM or 1 μM Auranofin (n = 3). **G** Flow cytometry to detect JC-1 staining of U251 and P3 treated with DMSO, 0.5 μM or 1 μM Auranofin (n = 3). FL2 corresponds to oxidized JC1 (JC1-Aggregate) and FL1 corresponds to non-oxidized JC1 (JC1 Monomers). **H** Transwell assays for LN229 and U251 treated with DMSO, 0.5 μM or 1 μM Auranofin (n = 3). **I** 3D tumor spheroid invasion assay of P3 treated with DMSO or 1 μM Auranofin (n = 3). Scale bar = 200 μm. **J** Representative images of HE staining of mouse brains from animals implanted with P3 and treated with DMSO, 5 mg/kg Auranofin, or 10 mg/kg Auranofin. Scale bar = 1 mm. **K** IHC to detect levels of the proliferation marker Ki67 in GBM xenografts. Scale bar = 100 μm. Data are shown as mean ± SEM. **P* < 0.05, ***P* < 0.01, ****P* < 0.001.

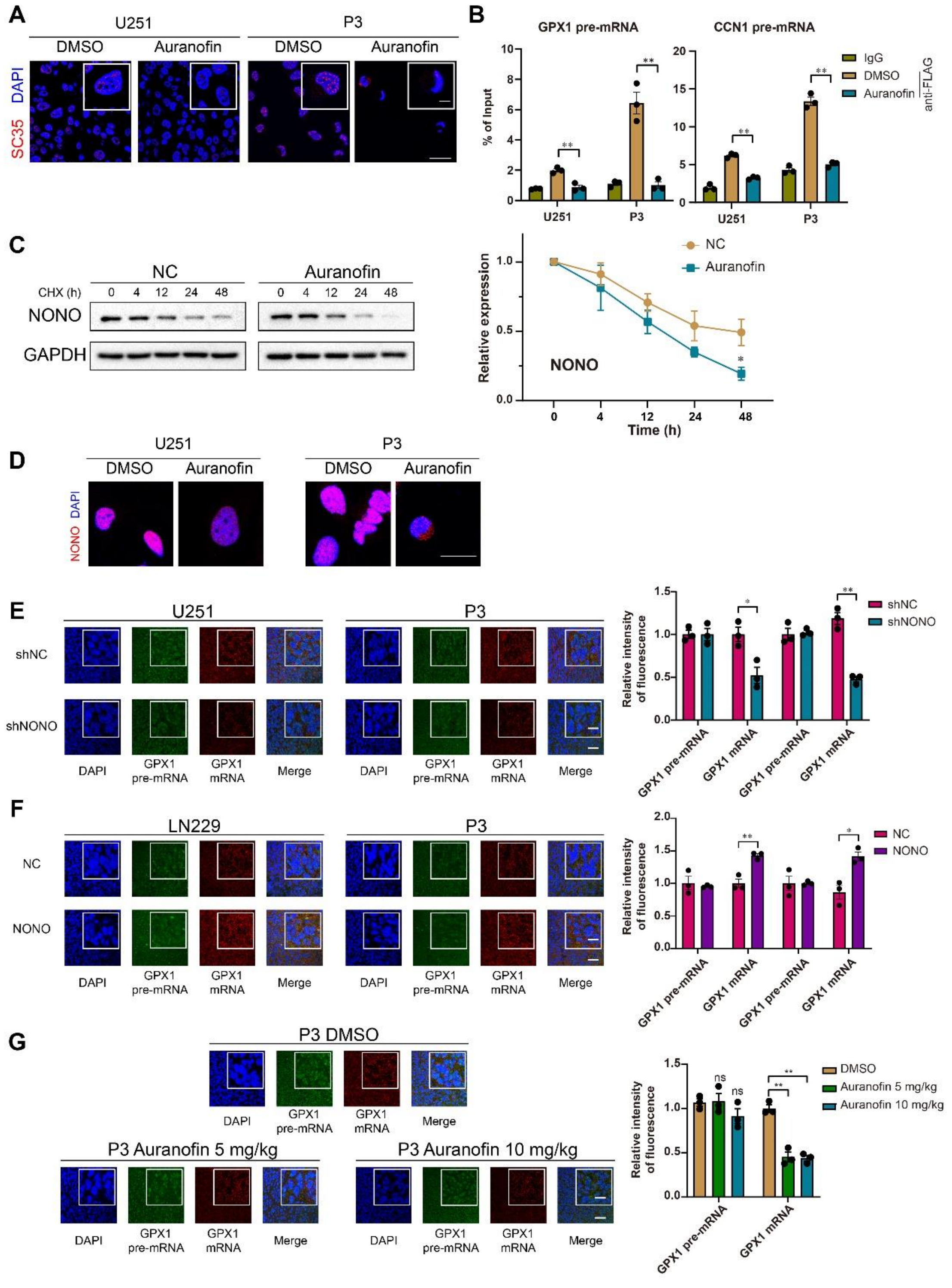

Figure S10. Auranofin impairs NONO stability and RNA binding, and inhibits NONO-mediated splicing in vivo.

A Immunofluorescence images for SC35 (red) and nuclear (blue) staining for U251 and P3 treated with DMSO or Auranofin (n = 3). Scale bar = 50 μm and 25 μm (magnified inset).

B RIP-PCR assay for detection of *GPX1* and *CCN1* pre-mRNA binding with NONO (n = 3). Input was used for normalization and IgG was used for the negative control. **C** Western blot to detect degradation of NONO in U251 treated with DMSO or Auranofin in CHX assay (n = 3). **D** Immunofluorescence staining showing subcellular localization of NONO (red) (n = 3). Nuclei are stained with DAPI (blue). Scale bar = 25 μm. **E-F** Fluorescence images for RNA FISH performed with probes for *GPX1* pre-mRNA or mRNA for detection in U251-, P3-, and LN229-shNONO or NONO-OE xenografts (n = 3). Scale bar = 50 μm and 10 μm (in magnification view). **G** Fluorescence images for RNA FISH performed with probes for *GPX1* pre-mRNA or mRNA for detection in P3-shNONO or NONO-OE xenografts treated with DMSO, 5 mg/kg or 10 mg/kg Auranofin (n = 3). Scale bar = 50 μm and 10 μm (magnified inset). Data are shown as mean ± SEM. $^*P < 0.05$, $^{**}P < 0.01$.

Table S1. The primer sequences used in the research

Gene symbol	Primer	Sequence
NONO	Forward	AACCTTCCCTGATGCGAGAG
	Reverse	TTCAGTTGTTGGTGGGGTCA
GAPDH	Forward	GCACCGTCAAGGCTGAGAAC
	Reverse	TGGTGAAGACGCCAGTGGA
CCNA2	Forward	AGCACTCTACACAGTCACGG
	Reverse	TGCTGTGGTGCTTTGAGGTA
CCNB1	Forward	GGCGAAGATCAACATGGCAG
	Reverse	TTTGCTTCCTTCTTCATAGGCA
CDK1	Forward	AGAAAGTGAAGAGGAAGGGGT
	Reverse	TAAGCACATCCTGAAGACTGACT
ZEB1	Forward	AATTCACAGTGGAGAGAAGCCA
	Reverse	GGTCGCCCATTCACAGGTAT
CD44	Forward	CGTGGAGAAAAATGGTCGCT
	Reverse	TGTGGGCAAGGTGCTATTGAA
SNAIL	Forward	CGGAAGCCTAACTACAGCGA
	Reverse	GCCAGGACAGAGTCCCAGAT
GPX1 pre-mRNA	Forward	AGGGCAAAATCCCGGTGAC
	Reverse	GAGCTTGGGGTCGGTCATAA
GPX1 mRNA	Forward	GTGCAACCAGTTTGGGCATC
	Reverse	GCCCACCAGGAACTTCTCAA
CCN1 pre-mRNA	Forward	AAGATGCTTGTGGTTTGGCCC
	Reverse	TTCCGTATGCGCTTTCGTTG
CCN1 mRNA	Forward	CAAGGGGCTGGAATGCAACT
	Reverse	TTCTCGTCAACTCCACCTCG
RPS9	Forward	CCGGAACAAACGTGAGGTCT
	Reverse	AAGGACGGGATGTTCACCAC
RPLP0	Forward	TTCTCTCGCCAGGCGTCC
	Reverse	AGCTGGGTTGTTTTCCAGGT
RPL3	Forward	ACCATTGTAAGACACCACCC
	Reverse	TCCTGCCCAAACACTTGGTTC
RPL21 (include 4th	Forward	AGGGAAAGAGGAGAGGCACC
intron)	Reverse	GTTTACAACAATGCCAACAGCA
RPL21(include 3rd	Forward	AGTTGTTCCTTTGGCCACATA
intron)	Reverse	TCAGGAAGCTATCTCGGCTC

Table S2. mRNA splicing protein list based on Gene Ontology

1	KHDRBS2	RBM17	POLR2B	M0QXG2	RBM25	DDX5	DNAJC8	BCAS2	YJU2	ISY1
11	U2AF1L5	CWF19L1	NOL3	TGS1	FIP1L1	CASC3	TFIP11	ZC3H10	REST	IK
21	WDR83	DHX9	GPATCH1	NCL	SNRPC	SPEN	BUD13	RBM3	RBM10	SRSF4
31	DDX41	USP4	RBFOX1	PQBP1	RBM42	RBM22	PRPF19	CDC40	SYNCRIP	LUC7L3
41	GEMIN7	WDR77	POLR2E	ZMAT2	SRSF2	DHX15	PPIE	NCBP2L	HNRNPA2B1	DHX16
51	CLNS1A	SNRNP200	NUP98	HTATSF1	LUC7L2	CPSF2	RSRC1	RBM41	PNN	PRMT7
61	QKI	U2SURP	RBM14	ESS2	SNRNP40	POLR2L	RBM8A	RBMXL1	LSM8	PTBP3
71	PRPF3	HNRNPR	POLR2J	POLR2H	HNRNPUL1	ZMAT5	RBM15B	STRAP	PRPF4	PPIL1
81	PUF60	PPWD1	DCPS	SRSF10	DDX20	PSIP1	SYF2	SLU7	PTBP2	ZBTB7A
91	PCBP4	CELF2	DHX8	LSM5	RBM7	DAZAP1	MTREX	PRPF40A	RBM5	TRA2B
101	LSM4	NUDT21	RBMX2	WBP11	THRAP3	LSM2	SNRNP35	AAR2	CD2BP2	NCBP2
111	MAGOHB	SF3B6	NSRP1	RBMXL2	WBP4	SF3B1	KHDRBS3	PLRG1	HNRNPM	SRSF3
121	LSM7	POLR2G	ZCRB1	DDX1	ALYREF	HNRNPC	CSTF3	RBM4B	SRPK3	YTHDC1
131	SNRPA	SMN1	SRSF7	PPIL3	RALY	CPSF3	SRRM4	DDX23	SF3B3	SCAF11
141	CELF1	NOVA2	ZCCHC8	CTNNBL1	POLR2D	SF3B2	RBM24	POLR2F	C9orf78	CCAR1
151	DHX38	RAVER2	STH	CWC22	NOVA1	METTL14	U2AF2	PRPF40B	EFTUD2	SART3
161	WTAP	SNRPB2	UBL5	SNIP1	CRNKL1	PRPF39	CELF4	POLR2A	DBR1	DYRK1A
171	SNRNP70	METTL3	PABPN1	SF1	K7EQG2	SNRNP27	M0R3G1	TXNL4A	HNRNPA3	GCFC2
181	CWC27	SAP18	SRRM2	PRPF6	SNRPN	DDX46	PTBP1	RNPS1	BUD31	CSTF2T
191	CELF3	DDX39A	DDX39B	METTL16	HNRNPL	RBM23	SNRPB	TRA2A	SRPK2	PRPF4B
201	M0R076	SNRPA1	HNRNPA1	SNRPGP15	FXR2	FXR1	SRRT	I3L521	MAGOH	RNF113A
211	SF3A3	SFSWAP	GEMIN2	SNUPN	FRG1	LUC7L	POLR2C	PRPF8	POLR2K	PHF5A
221	HNRNPK	SETX	DDX17	USP49	C1QBP	SNW1	CPSF7	PABPC1	TXNL4B	GEMIN8
231	GEMIN5	PDCD7	KHDRBS1	MBNL1	RNPC3	WDR33	RBM11	SRSF8	MBNL3	NCBP1
241	PRMT5	SRSF1	CSTF2	CPSF1	HNRNPF	CWF19L2	SMU1	RBMY1B	SFPQ	SON
251	HMX2	PCF11	UPF3B	PRPF18	SRSF11	COIL	TIA1	CACTIN	RBMX	SF3A1
261	FAM172A	SF3A2	SF3B4	RBMY1F	POLR2I	RBM15	RBM6	RBMXL3	LARP7	HNRNPU
271	RBM4	SCNM1	KHDC4	SF3B5	CELF5	SRRM1	NONO	RAVER1	PRCC	JMJD6
281	U2AF1L4	GPKOW	PCBP2	PCBP1	PSPC1	SRSF12	GEMIN6	ELAVL1	PAPOLA	PRPF31
291	DDX42	SUGP1	CHERP	XAB2	MFAP1	PPIH	EIF4A3	CDC5L	HNRNPH1	HNRNPH3
301	SYMPK	CSTF1	CLP1	HSPA8	ZRSR2	ZRSR2P1	USP39	HNRNPD	MYOD1	FUS
311	ELAVL2	PRDX6	GTF2F2	HNRNPH2	SNU13	GTF2F1	ESRP2	CWC15	SNRNP48	KDM1A
321	FMR1	AQR	DHX35	SLC39A5	SRSF6	SRSF5	SRSF9	SART1	SNRNP25	SRPK1
331	CWC25	GEMIN4	SNRPD3	SNRPD2	SNRPD1	LSM6	LSM3	SNRPG	SNRPF	SNRPE
341	CELF6	RBM39	PRPF38A	YBX1	U2AF1	MBNL2	CPSF4	SMNDC1	RBM19	RBFOX2
351	HNRNPA0	RBFOX3	RBMY1A1	CIRBP	CDK13					

Table S3. The genes regulated by NONO both in U251 and P3 cell lines

Gene Symbol	Log2 transformed expression in GBM[a]	Log2 (fold change)	*P* value	Description
GPX1	13.86	-1.61	0.00	Glutathione peroxidase 1
CCN1	11.80	-1.58	0.00	Cysteine-rich, angiogenic inducer, 61
AK4	11.66	-1.49	0.00	Adenylate kinase 4
NKTR	11.21	-1.24	0.00	Natural killer cell triggering receptor
GFPT2	10.97	-1.54	0.00	Glutamine-fructose-6-phosphate transaminase 2
LONRF2	10.55	-2.24	0.00	LON peptidase N-terminal domain and ring finger 2
NPNT	10.33	-1.50	0.00	Nephronectin
KIT	8.60	-1.00	0.02	V-kit Hardy-Zuckerman 4 feline sarcoma viral oncogene homolog
DCHS2	7.00	-1.14	0.01	Dachsous cadherin-related 2
STON1-GTF2A1L	2.17	-3.20	0.00	General transcription factor IIA, 1-like

a: Log2 transformed expression in GBM: The mean log2-transformed expression level in GBM based on TCGA database.

Table S4. The splicing efficiency change of GPX1 and CCN1.

SYMBOL	NM_id	Intron number	5' or 3' splicing site	siNC spliced reads	siNONO spliced reads	siNC unspliced reads	siNONO unspliced reads	siNC splicing efficiency	siNONO splicing efficiency	FC[a] siNONO vs siNC	Regulation
GPX1	NM_000581.4	intron1	3ss	2084	915	17	44	122.59	20.8	0.17	down
CCN1	NM_001554.5	intron3	3ss	940	627	13	14	72.31	44.79	0.62	down
CCN1	NM_001554.5	intron3	5ss	940	625	10	9	94	69.44	0.74	down
CCN1	NM_001554.5	intron4	3ss	1102	820	10	15	110.2	54.67	0.5	down

a: FC: Fold Change
